# A Water‐Soluble PVA Macrothiol Enables Two‐Photon Microfabrication of Cell‐Interactive Hydrogel Structures at 400 mm s^−1^

**DOI:** 10.1002/adma.202510834

**Published:** 2026-01-08

**Authors:** Wanwan Qiu, Margherita Bernero, Muja Emilie Ye, Xianjun Yang, Philipp Fisch, Ralph Müller, Xiao‐Hua Qin

**Affiliations:** ^1^ Institute for Biomechanics ETH Zurich Zurich Switzerland; ^2^ Bringing Materials to Life Initiative ETH Zurich Zurich Switzerland

**Keywords:** 3D printing, biomaterials, hydrogels, polyvinyl alcohol, thiol‐ene reactions, two‐photon polymerization

## Abstract

Two‐photon polymerization (2PP) has garnered increasing attention for engineering hydrogels with tailored architectures and controlled cellular responses. However, current 2PP strategies typically rely on (meth)acrylated proteins and inefficient chain‐growth crosslinking mechanisms. Although thiol‐ene photo‐click reactions can enhance 2PP efficiency, commercial water‐soluble thiol crosslinkers (e.g., DTT—dithiothreitol) tend to form intramolecular loops and introduce structural defects due to their short molecular length. As a result, high polymer concentrations (often up to 20%–50%) are required to achieve satisfactory print fidelity. Here, we develop a series of water‐soluble, polyvinyl alcohol macromolecular thiol (PVASH, bearing 10–35 thiol groups) for fast high‐fidelity hydrogel microfabrication via 2PP. A two‐step synthesis yields PVASH with tunable degrees of substitution and excellent water‐solubility. Compared to DTT and polyethylene glycol di‐thiol, PVASH‐based hydrogels exhibit reduced swelling, enhanced mechanical properties, and significantly improved printing fidelity. Notably, several complex hydrogel structures are fabricated at laser power as low as 20 mW and high scanning speeds of up to 400 mm s^−1^, achieving sub‐micron feature size at 3% polymer concentration. After biofunctionalization with RGD motifs, the micro‐scaffolds support cell infiltration, adhesion, proliferation, and osteogenic differentiation. Altogether, this work reports a new strategy for 2PP microfabrication of cell‐interactive hydrogel structures with unprecedented printing efficiency and precision.

## Graphical abstract

A PVA‐based macromolecular thiol‐ene formulation enables efficient two‐photon polymerization at extremely low polymer concentrations and high writing speeds of 400 mm s^−1^ (20×), allowing high‐fidelity laser writing of cell‐interactive hydrogel structures on demand.

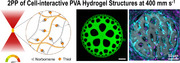

## 1 Introduction

Two‐photon polymerization (2PP) is a direct laser writing technique for fabricating high‐resolution 3D hydrogel architectures in tissue engineering and drug delivery [1, 2, 3, 4]. This 3D printing technology enables the creation of user‐dictated hydrogel structures with (sub)‐micron‐scale resolution [5, 6]. 2PP utilizes femtosecond laser pulses to induce localized solidification within a photosensitive precursor solution, allowing for the direct writing of complex 3D architectures [7, 8, 9, 10]. The nonlinear nature of two‐photon absorption confines the photochemical reaction to the focal volume of the laser, providing superior spatial control compared to conventional single‐photon photolithography techniques.

Although 2PP has shown the promise to fabricate extracellular matrix (ECM)‐mimicking hydrogels, it poses several challenges in selecting suitable base materials [11]. The key challenge lies in combining biocompatibility, biodegradability, water‐solubility, and high photoreactivity within a single formulation for effective 2PP. Recent advances have witnessed the development of water‐soluble two‐photon photoinitiators [12, 13], cell‐compatible hydrogel precursors such as gelatin vinyl esters [14], and novel (de)crosslinking mechanisms [15]. Notably, Li et al. reported a series of water‐soluble, cyclic benzylidene ketone‐based photoinitiators that have large two‐photon absorption cross sections (> 100 GM, 1 GM = 10^−50^ cm^4^ s photon^−1^) [12]. The use of P2CK enabled 2PP of polyethylene glycol diacrylates (PEGDA, *M_w:_* 700 Da) hydrogel scaffolds with 50% water content [12, 16]. Although PEGDA are highly reactive, the irritancy and toxicity of acrylate groups are problematic for cell culture applications. Qin et al. developed vinyl ester derivatives of gelatin and hyaluronan (HA) to circumvent the toxicity issues of PEGDA [14, 17, 18]. The rate of chain‐growth polymerization of vinyl esters is moderate; however, their co‐polymerization with thiols can significantly improve the photoreactivity to the level of acrylates [19, 20]. Compared to di‐tyrosine crosslinking [21, 22], radical‐mediated thiol‐ene click reactions allow faster crosslinking as one thiyl radical can be reused for many times during the crosslinking [23, 24, 25, 26].

**SCHEME 1:**
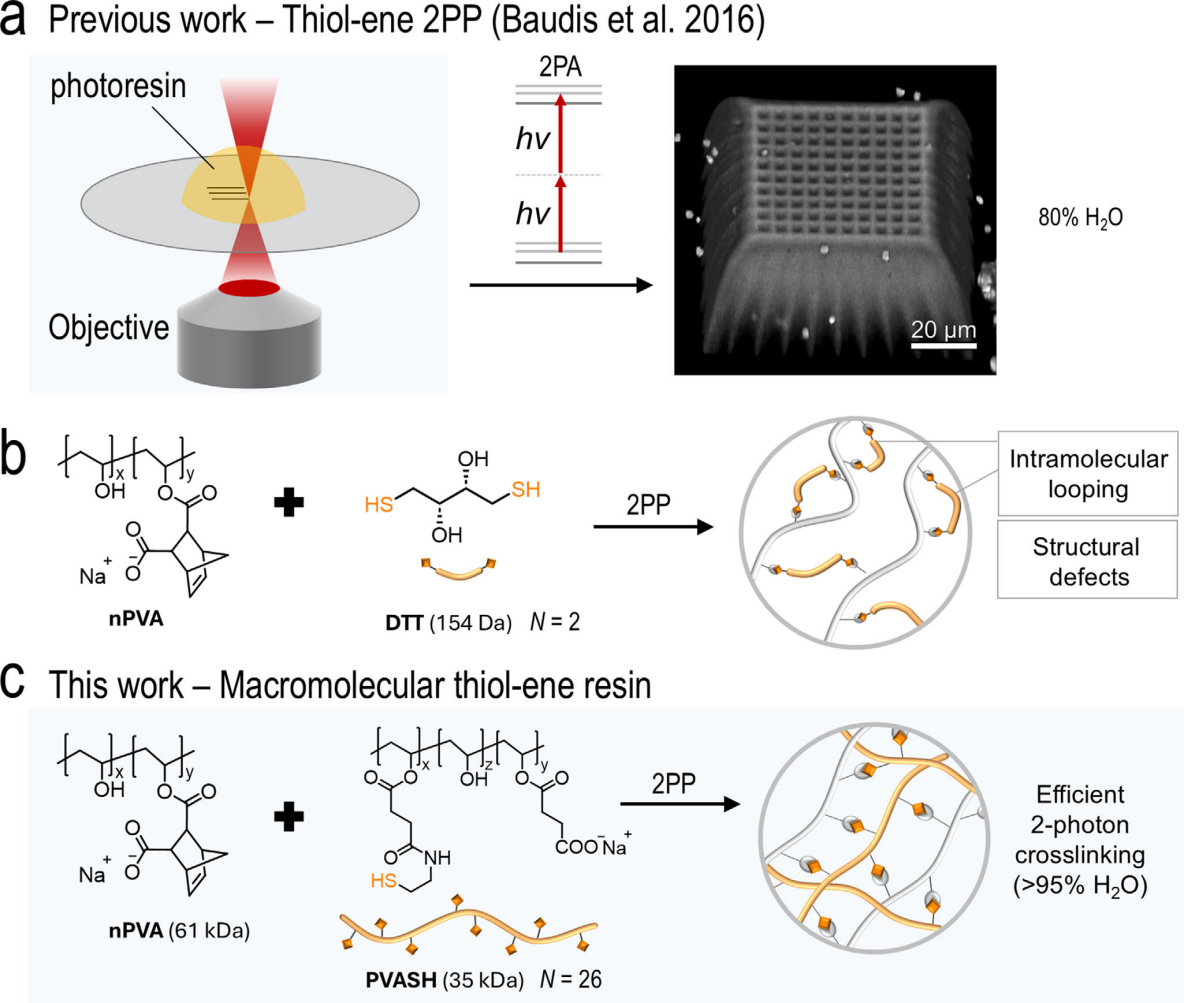
(a) Schematic of direct laser writing of 3D microstructures by 2PP. A representative 3D scaffold fabricated using a reference formulation (20% PVA) showing pronounced swelling behavior [31]. (b) A schematic of the crosslinking of nPVA with DTT crosslinker, which tends to introduce ineffective loops within the hydrogel network at low polymer concentrations. (c) Schematic of this work demonstrating the efficient crosslinking of nPVA with a macromolecular PVASH crosslinker via two‐photon‐initiated thiol‐ene reaction. A tunable degree of substitution (*N* = 10–35) is achievable in the synthesis of PVASH.

One key consideration in the fabrication of thiol‐ene hydrogels is the selection of water‐soluble thiols, such as dithiothreitol [24] (DTT, *N* = 2, *M_w_*_:_ 154 Da) and PEG di‐thiols [27, 28] (PEG2SH, *N* = 2, *M_w_*_:_ 2–5 kDa). However, these crosslinkers have a limited number of reactive groups. DTT tends to react with the same polymer backbone during thiol‐ene crosslinking, especially at low polymer concentrations. This leads to the formation of primary loops [29, 30], a type of defect caused by intramolecular crosslinking (Scheme 1a,b), which reduces network connectivity and compromises gel stability. This challenge becomes particularly significant in high‐precision microfabrication of soft ECM‐like hydrogels via 2PP, where low polymer concentration is key to mimic the low mechanical stiffness of native tissues. Table S2 provides a comparison of state‐of‐the‐art 2PP systems. To date, most 2PP strategies rely on high polymer concentrations (20%–50%) to achieve printability at low writing speeds.

**FIGURE 1:**
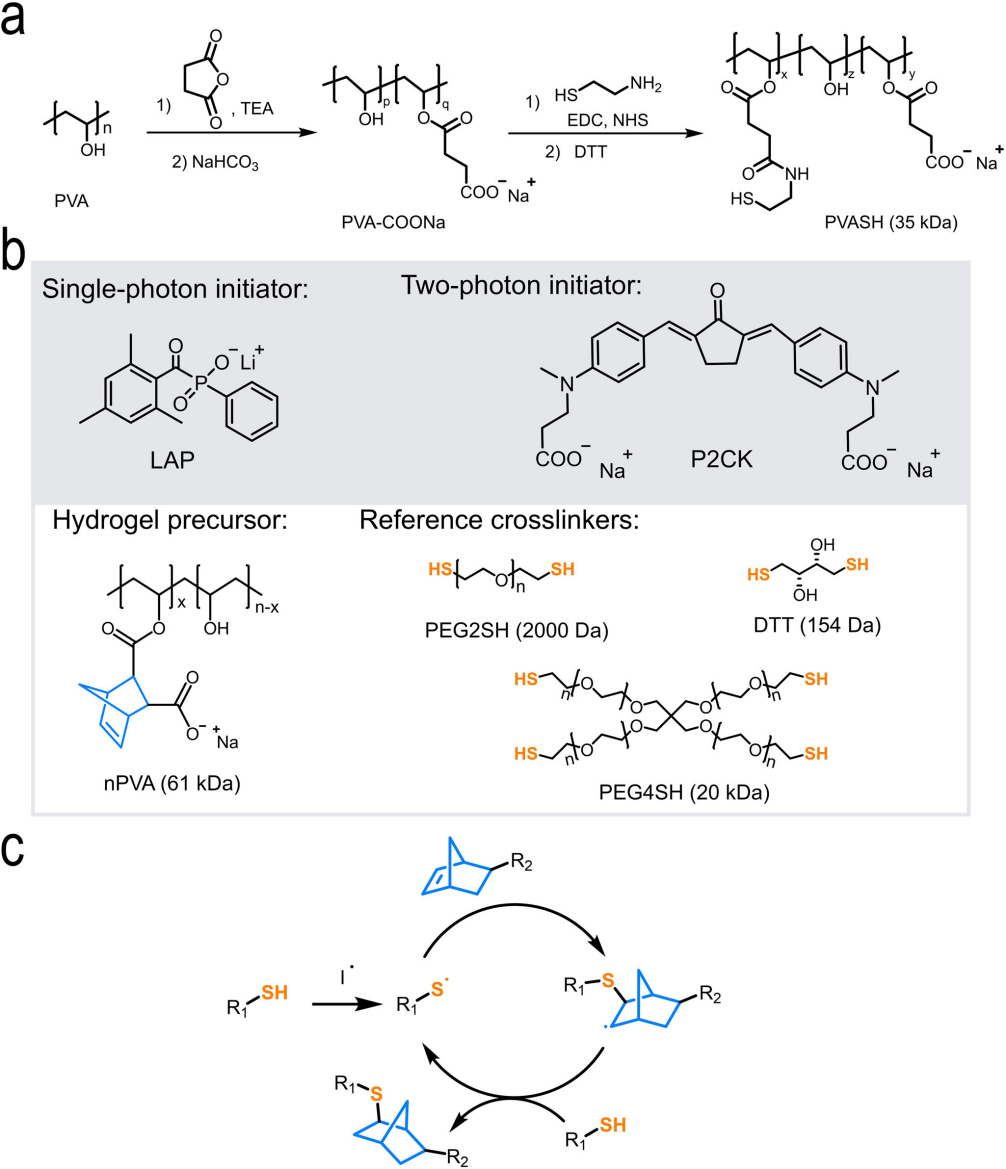
(a) Synthetic route of sodium salt of thiol‐functionalized PVA (PVASH): Sodium carboxylate side chains were introduced into PVA to provide reactive sites for thiol functionalization and to enhance water‐solubility. DTT was added to cleave any formed disulfide bonds into free cysteines. (b) Chemical structures of water‐soluble photoinitiators (LAP, P2CK), the hydrogel precursor (nPVA), and reference thiol crosslinkers (DTT, PEG2SH, and PEG4SH). (c) Proposed mechanism of radical‐mediated thiol‐norbornene photoclick reaction.

Herein, we present the design, synthesis, and functionalization of a macromolecular PVA thiol crosslinker (PVASH) for fast 2PP microfabrication of cell‐interactive hydrogels. We reason that using a macromolecular thiol crosslinker can effectively form a stable hydrogel structure via 2PP even at low polymer concentrations, offering greater flexibility and design freedom for high‐fidelity hydrogel microfabrication (Scheme 1c). We devised a two‐step process to synthesize the PVASH linker with both a sufficient number of thiol groups and good water‐solubility (Figure 1a). By mixing PVASH with norbornene‐functionalized PVA (nPVA) and photoinitiators, we formulated a series of highly efficient mixtures and investigated their physicochemical properties as a function of polymer concentration and crosslinker architecture. In comparison to the commonly used DTT and PEG2SH crosslinkers, our PVASH features multiple reactive groups (*N* > 10), which significantly enhances printability during 2PP. Furthermore, we demonstrate that the printed micro‐scaffolds can be biofunctionalized for cell culture and tissue engineering applications.

## 2 Results and Discussion

### 2.1 Design of PVASH Crosslinker

In this study, we designed a series of PVASH that can form elastically active networks at low polymer concentrations during 2PP. Commercial pristine PVA bears a high number of hydroxyl groups, ranging from 200 to 2000, depending on the *M_w_*. Compared to PEG, PVA is a linear polymer and offers more flexibility in chemical modifications. Although PVA is a biocompatible polymer, its dissolution in water typically necessitates heating due to H─bonds. One elegant approach to improve its water‐solubility is to introduce sodium carboxylate groups through the reaction of PVA with anhydrides. For example, nPVA has been synthesized with excellent water‐solubility. Biofunctionalized nPVA hydrogels have been used as hemostatic materials [32], cell‐responsive hydrogels [33], and volumetric bioprinting [34].

Baudis et al. [31], reported a one‐step method to synthesize PVASH by the conversion of PVA with γ‐thiobutyrolactone in dry DMSO for 3 days. Although the degree of substitution (DS) reached up to 6%, the crosslinking efficiency of PVASH was inferior to DTT, presumably due to the poor water‐solubility as a result of the hydrophobicity of thiol groups. To address this, we devised a two‐step approach. PVA was first modified with sodium carboxylate through reaction with succinic anhydride. After dialysis and neutralization, sodium carboxylate of PVA (PVA‐COONa) was obtained with a DS of ∼10%. Some of these carboxylate groups were then used to graft thiol groups by reacting with cysteamine. Importantly, the remaining sodium carboxylate groups enhance the macromer's water‐solubility. Compared to the previously reported method [31], our reaction conditions are milder and offer greater control by adjusting the molar ratio of the reactants. The DS of both PVA‐COONa and PVASH were confirmed via ^1^H‐NMR (Figures S13 and S14). By mixing nPVA [33, 34] and PVASH, we formulated a dual PVA mixture that can be photo‐crosslinked via one‐photon or two‐photon excitation using either LAP (lithium phenyl‐2,4,6‐trimethylbenzoylphosphinate, one‐photon) [35], or P2CK [12] (two‐photon) as the photoinitiator (Figure 1b).

**FIGURE 2:**
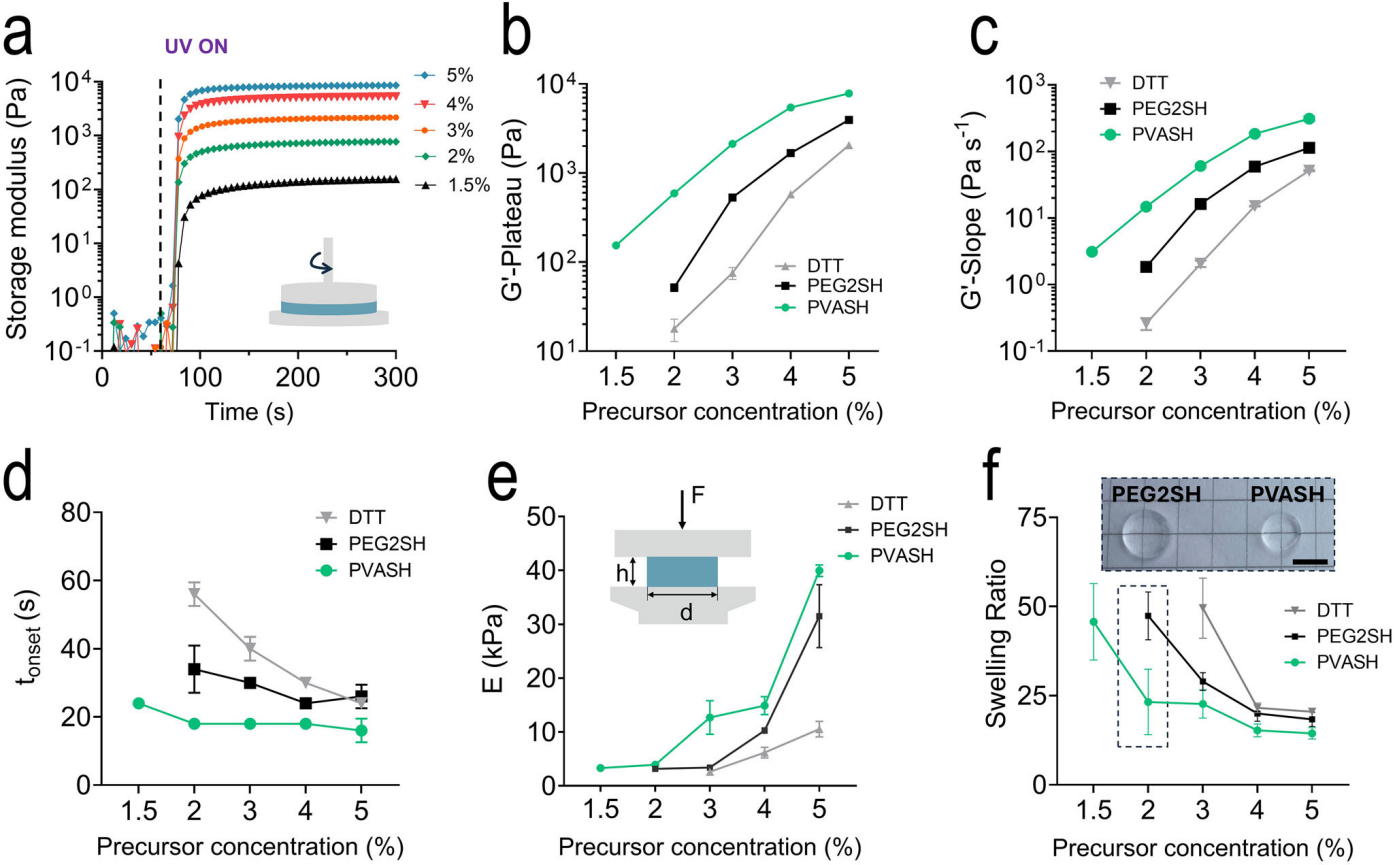
(a) Time sweep of storage moduli (G′) of PVASH hydrogels at varying precursor concentrations (sum of all components) and a thiol‐ene ratio of 1:1 with nPVA. Light intensity: 10 mW/cm^2^; LAP concentration: 0.05%. (b–d) G′‐plateau (b), G′‐slope (c), and *t*_onset_ (d) as an influence of precursor concentration and crosslinker. Data represented as mean ± SD (n = 3). *t*_onset_ is defined at the first data point where G′ > 5 Pa. The DTT group fails to crosslink at 1.5%. (e) Compressive modulus (*E*) as a function of hydrogel precursor concentration and crosslinker. Data represented as mean ± SD (n = 3). PEG2SH and DTT groups cannot form stable gels suitable for testing at 1.5% and 2%, respectively. (f) Equilibrium mass swelling ratio as a function of hydrogel precursor concentration. Data represented as mean ± SD (n = 3). Insert: image of PEG2SH and PVASH hydrogel samples (2%) showing their physical appearance. An enlarged version of the image is provided in the Supporting Information. Scale bar, 5 mm.

We tested the thiol‐ene crosslinking of PVA hydrogels (1:1 thiol‐ene stoichiometric ratio) using in situ photo‐rheology to evaluate the influence of different thiol crosslinkers. Two commercial di‐thiol crosslinkers (DTT and PEG2SH) and one tetra‐thiol crosslinker (PEG4SH, *N* = 4, *M*_w__:_ 20 kDa), were used as the references. The storage moduli (G′) of PVASH hydrogels increased with macromer concentration due to a higher concentration of reactive groups, spanning a broad range from 0.1 to 10 kPa (Figure 2a). The PVASH group showed the highest G′‐plateau, exceeding those of the reference groups at equivalent precursor concentrations (Figure S1). Specifically, the G'‐plateau of the PVASH group increased from 0.16 kPa at 1.5% polymer concentration to 7.8 kPa at 5%. In comparison, the PEG2SH group showed a G′‐plateau of 0.05 kPa at 2% and 3.9 kPa at 5%, while the DTT group exhibited a G′‐plateau of 0.018 kPa at 2%, increasing to 2.1 kPa at 5% (Figure 2b; Table 1). We compared the slope of G′ (G′‐slope) as a measure of the crosslinking rate. The PVASH group exhibited a significantly higher G′‐slope compared to DTT and PEG2SH, particularly at precursor concentrations below 3% (Figure 2c). Moreover, the gelation onset time (*t*_onset_), defined as the first time point at which G′ exceeds 5 Pa, was evaluated for the different formulations (Figure 2d). PVASH showed lower t_onset_ values than DTT and PEG2SH at precursor concentrations of 1.5%–3%. This difference is attributed to the distinct molecular architectures of the thiol crosslinkers. The macromolecular structure of PVASH enables more efficient crosslinking with nPVA and reduces the likelihood of primary loop formation [36]. In contrast, DTT and PEG2SH exhibit a concentration‐dependent crosslinking rate and *t*_onset_. These findings underscore the critical influence of crosslinker architecture on the thiol‐ene photocrosslinking of PVA hydrogels. Interestingly, the commercial tetra‐thiol crosslinker (PEG4SH) exhibited a similar efficiency to PVASH (Figure S1). However, it is important to note that PEG4SH contains only four pendant thiol groups, which may limit its fexibility for RGD functionalization compared to PVASH (ca. 26 thiol groups).

**TABLE 1:** Physicochemical properties of PVA hydrogels as a influence of different thiol linkers and total polymer concentrations. Thiol‐ene ratio: 1:1. Light intensity: 10 mW/cm^2^.

	Concentration (%)^a^	Storage modulus *G′* (kPa)^b^	Loss modulus *G′′* (Pa)^b^	Gelation time (s)	Compressive modulus *E* (kPa)	Swelling ratio *Q_m_*	Thiol concentration^c^
PVASH (35 kDa)	1.5%	0.16 ± 0.002	0.26 ± 0.14	12.3 ± 0.2	3.3 ± 0.07	54.7 ± 10.7	0.74
	2%	0.6 ± 0.01	0.5 ± 0.18	9.4 ± 2.2	3.9 ± 0.15	23.2 ± 9.2	
	3%	2.2 ± 0.069	1.43 ± 0.82	9.3 ± 4.7	12.7 ± 3.12	22.7 ± 4	
	4%	5.5 ± 0.29	4.7 ± 0.4	6.5 ± 4.7	14.9 ± 1.7	15.3 ± 1.78	
	5%	7.84 ± 0.63	6.44 ± 2.31	7.2 ± 0.0	39.9 ± 1.1	14.4 ± 1.57	
PEG2SH (2 kDa)	2%	0.053 ± 0.006	0.56 ± 0.25	30 ± 8.1	3.16 ± 0.3	47.4 ± 6.7	1
	3%	0.53 ± 0.036	0.34 ± 0.14	16.2 ± 0.0	3.4 ± 0.4	29.0 ± 2.5	
	4%	1.67 ± 0.12	0.81 ± 0.03	15.8 ± 3.9	10.3 ± 0.5	20.0 ± 2.2	
	5%	3.94 ± 0.06	1.91 ± 1.07	13.5 ± 6.3	31.5 ± 5.9	18.3 ± 2.0	
DTT (154 Da)	2%	0.018 ± 0.006	0.32 ± 0.25	21.5 ± 3.15	—	—	13
	3%	0.076 ± 0.014	0.27 ± 0.27	29.0 ± 1.90	2.62 ± 0.07	49.5 ±8.44	
	4%	0.58 ± 0.012	0.69 ± 0.44	19.0 ± 7.35	6.18 ± 0.99	21.6 ± 1.35	
	5%	2.08 ± 0.046	1.20 ± 0.45	18.0 ± 0.02	10.5 ± 1.44	20.5 ± 0.67	
PEG4SH (20 kDa)	1.5%	0.17 ± 0.02	0.36 ± 0.25	15.0 ± 2.2	—	—	0.2
	2%	0.5 ± 0.05	0.6 ± 0.2	14.0 ± 0.5	—	—	
	3%	2.7 ± 0.04	0.7 ± 0.4	11.0 ± 0.8	—	—	
	4%	6.2 ± 0.3	1.7 ± 1.3	7.0 ± 1.4	—	—	
	5%	10.4 ± 0.07	5.1 ± 2	7.7 ± 2.4	—	—	

^a^ The total polymer concentration in the formulation.

^b^ Plateau values after in situ photocrosslinking (one‐photon, 365 nm) for 300 s.

^c^ The number of thiol groups per 1000 Da of the crosslinkers.

We further validated the photo‐rheology findings through unconfined compression mechanical testing and swelling analysis of the different hydrogels (Figure 2e). The PVASH group exhibited significantly higher compressive moduli compared to the reference materials. Notably, the DTT group cannot form stable hydrogels suitable for compression testing at precursor concentrations below 3%, while the PEG2SH group required a minimum concentration of 2%. These results highlight the advantages of PVASH in enabling efficient photocrosslinking and the formation of stable hydrogels at low polymer concentrations. We investigated the mass swelling ratio (Figure 2f) as an indicator of defects in the network structure. The swelling ratio decreases as the increase of polymer concentration. At the same concentration, the PVASH group showed the lowest swelling ratio. The swelling ratios of the DTT and PEG2SH groups at 3% were 49.5 ± 8.44 and 29.0 ± 2.75, respectively, while the PVASH group showed a significantly lower swelling ratio of 22.7 ± 4 (Table 1). The volumetric difference between the 2% PEG2SH and PVASH hydrogels is shown in Figure 2f (top) and Figure S2. Further, Ellman's assay was employed to evaluate thiol conversion by quantifying the relative amount of thiol groups before and after crosslinking. PEG2SH and PVASH exhibited comparable conversion, both exceeding 85% at polymer concentrations of 2% and 4% (Figure S3, Table S1). In contrast, DTT showed substantially lower conversion at 4% polymer concentration (∼76%). These results indicate that PEG2SH and PVASH undergo efficient and comparable thiol conversion. When considered alongside the compressive modulus and swelling ratio measurements, the data further suggest the presence of structural defects within the hydrogel network of the PEG2SH group, particularly at low polymer concentration (2%).

**FIGURE 3:**
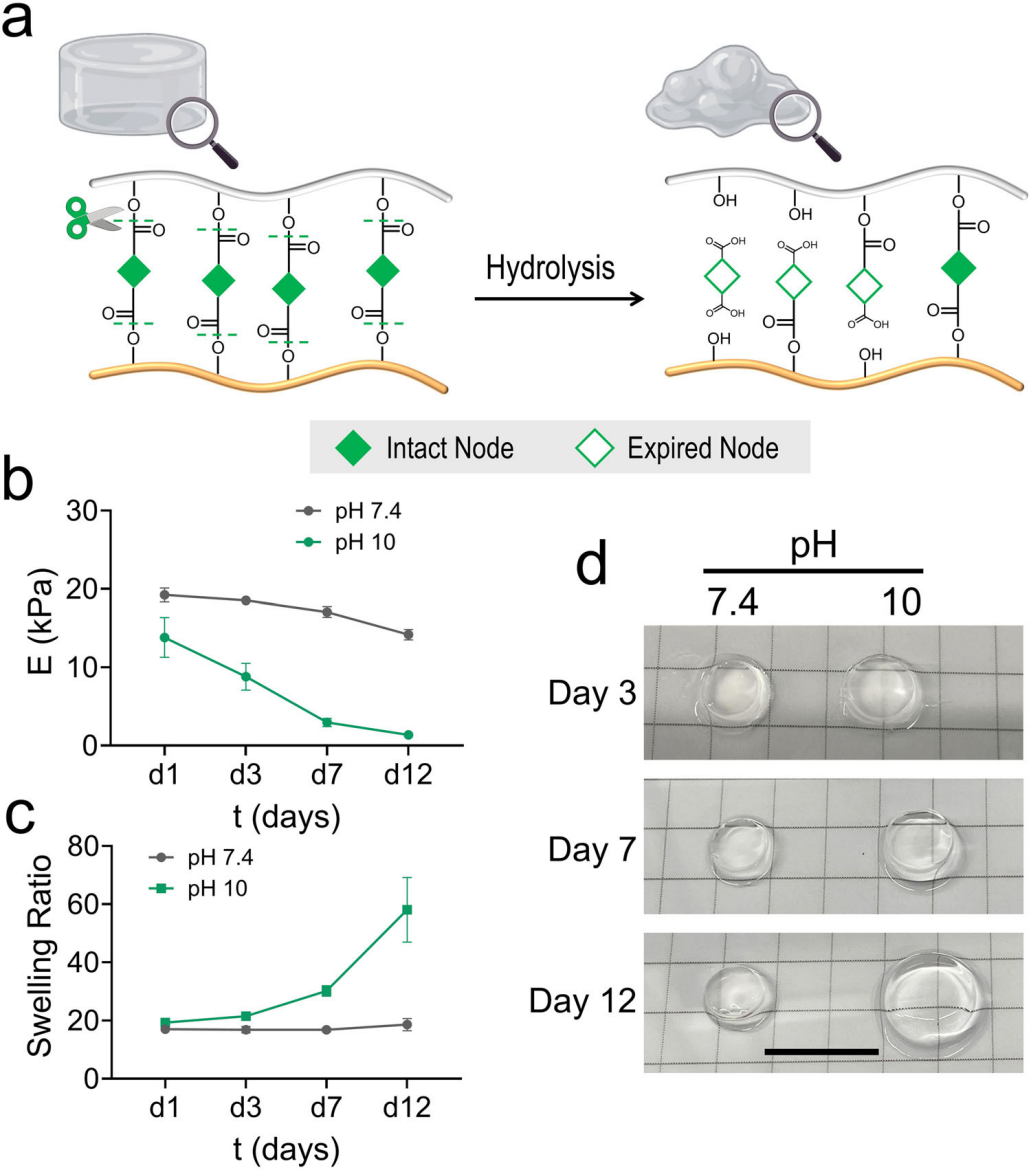
(a) Schematic of the hydrolytic degradation of PVA hydrogels. (b,c) Compressive modulus (*E*) (b) and mass swelling ratio (c) of 4% PVA hydrogels after incubation in 1 × PBS (pH 7.4 or pH 10) at 37°C over time. Complete degradation occurred in the pH 10 buffer after 14 days. Data are presented as mean ± SD (*n* ≥ 3). (d) Representative images showing the physical appearance of hydrogels after incubation in PBS (pH 7.4 or 10) at 37°C. Scale bar, 1 cm. Schematics in (a) created using BioRender.com.

Considering the presence of ester linkages in the gel precursors (Figure 3), we assessed the hydrolytic stability of PVASH hydrogels incubated in PBS (pH 7.4 or 10) at 37°C. Degradation was evaluated by monitoring changes in compressive modulus and mass swelling ratio over time. The compressive modulus decreased under both conditions. At pH 10, the modulus dropped sharply from ∼14 to ∼1.3 kPa within 12 days (Figure 3b), whereas at pH 7.4 it declined more modestly from ∼19 to ∼14 kPa, with negligible changes in swelling ratio (Figure 3c). At pH 10, the swelling ratio increased dramatically from ∼19 on day 1 to ∼60 on day 12, accompanied by a loss of shape fidelity (Figure 3d) and complete degradation by day 14, reflecting accelerated hydrolysis of ester groups within the network. These results demonstrate that PVASH hydrogel degradation is strongly pH‐dependent: the gels maintain structural integrity under neutral conditions but undergo fast degradation under alkaline conditions.

### 2.2 Two‐Photon Microfabrication

**FIGURE 4:**
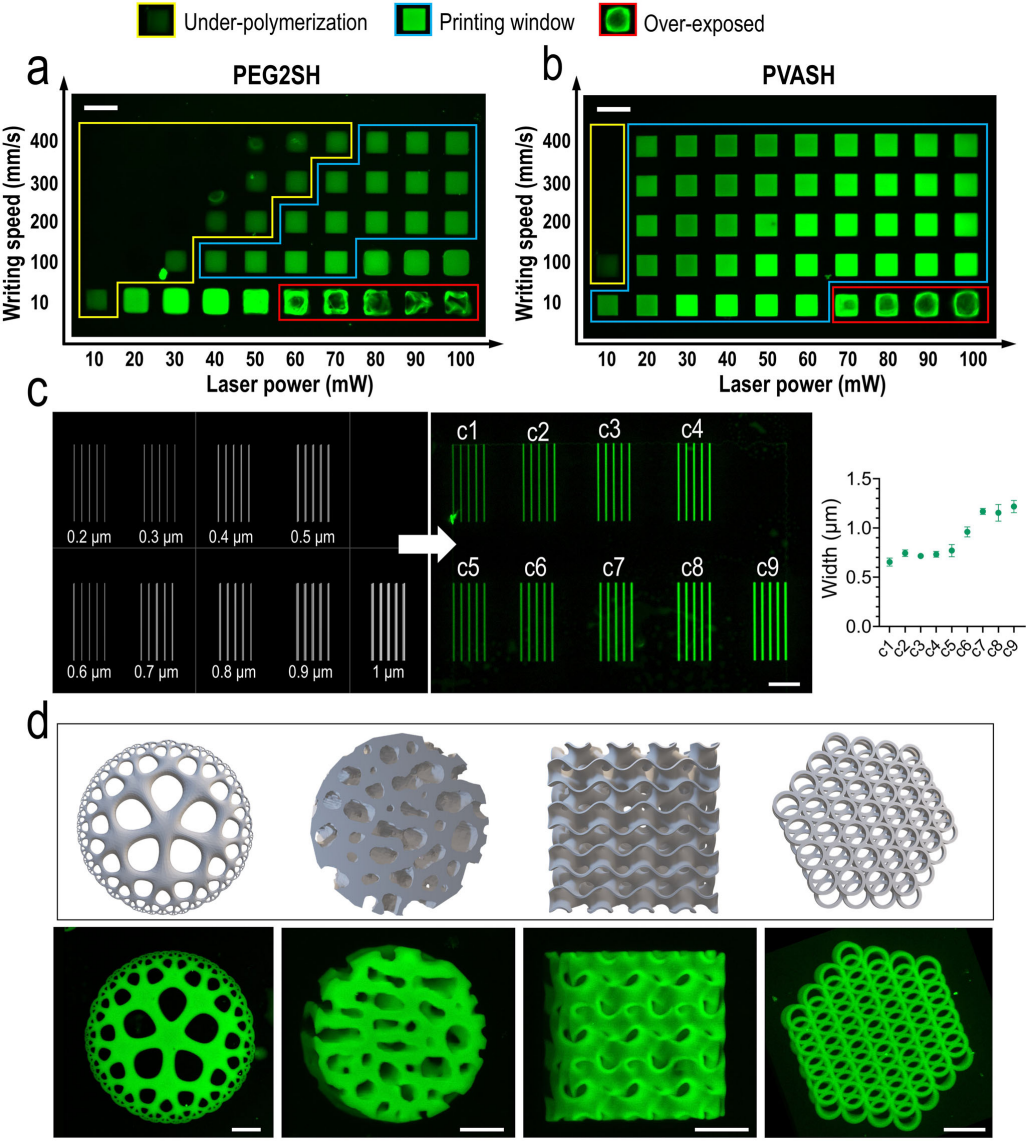
(a,b) Processing window of 4% formulations with either PEG2SH (a) or PVASH (b) as the crosslinker at varying laser power and scanning speeds with a 20× objective. Incomplete structures with edge sagging due to insufficient polymerization (yellow). Well‐defined structures with good fidelity (blue). Structures damaged by overexposure (red). Scale bars, 50 µm. (c) Printing line models with different width (left) and confocal images (right) of the polymerized lines fabricated using the 4% PVASH group with a 40 × oil‐immersion objective. Confocal imaging was performed using a 63 × oil‐immersion objective. Scale bar, 20 µm. (d) List of STL models (top) and confocal microscopic images (bottom) of the printed microstructures, including: Echinodermania [38] (300 × 300 × 29 µm^3^), femur (200 × 200 × 125 µm^3^), gyroid (150 × 150 × 150 µm^3^), and hexagonal (230 × 200 × 30 µm^3^). The microstructures were printed with 4% PVASH at writing speeds of 300 mm s^−1^ and laser power of 70 mW (20 × objective). Scale bars, 50 µm.

We investigated the processing window of PVASH for two‐photon microfabrication of hydrogel structures (Figure 4) using P2CK [12] as the photoinitiator. The range of optimal processing parameters that allow for reproducible fabrication of microstructures is defined as the “processing window” [37]. Outside this window, the formulation is either insufficiently cured or over‐cured. Figure 4a,b presents a comparative analysis of the processing window of PEG2SH and PVASH groups (4%), where the laser power (10–100 mW) and scanning speeds (10–400 mm s^−1^) were varied. The PVASH group exhibited a significantly larger processing window compared to PEG2SH. Notably, despite its macromolecular nature, only a very few well‐defined structures were obtained with the commercial PEG4SH (Figure S4). In contrast, printing with DTT was unsuccessful under the same set of parameters. To assess the mechanical properties of printed microstructures, we performed nanoindentation on 4% PVASH and 4% PEG2SH hydrogels. For direct comparison between one‐photon UV crosslinking and 2PP, cast gels of the same formulations were tested under identical conditions on the same instrument. Two‐photon printing largely preserved the bulk stiffness of 4% PVASH, whereas 4% PEG2SH showed ∼50% lower stiffness than its cast control (Figure S5). These findings highlight the robustness of PVASH for two‐photon microfabrication and emphasize the critical role of the crosslinker's molecular architecture in determining mechanical retention under 2PP. Compared with thiolated hyaluronic acid (HASH) and thiolated gelatin (GelSH), which are inherently bioactive and contain cell‐adhesive motifs, PVASH provides a fully synthetic alternative with greater design flexibility. In particular, thiol–norbornene photoclick PVA hydrogels allow near‐independent tuning of biochemical functionalization [33], and mechanical properties.

Notably, when the polymer concentration was reduced to 3%, the PVASH group remained printable in a large processing window, while the PEG2SH group became poorly printable (Figure S6, Movie S1). When the polymer concentration decreased to 2%, the printability declined significantly (Figure S7a) because of the insufficient crosslinking density. Drawing on the double‐network concept in previous work [34, 39], we hypothesized that incorporating a sacrificial network could enhance the printability at low polymer content during 2PP. By adding 3% gelatin, we successfully improved the printability of 2% PVASH formulation with a processing window comparable to that of 4% PVASH formulation. The printed structures remained stable after two days of incubation at 37°C (Figure S7b). More than 80% of the gelatin can diffuse out based on a previous report [34]. Nevertheless, future work is warranted to investigate the gelatin release from hydrogel structures by 2PP.

Next, we used the 40 × objective to fabricate thinner lines (0.2–1 µm) to further evaluate the printability of the PVASH formulations. Due to the confocal pixel size limit (ca. 0.23 µm), features below this threshold could not be quantified with high accuracy. Measured linewidths for designs ≤ 0.6 µm therefore represent upper bounds. Consequently, the apparent widths of the finest lines converged to ∼0.6–0.8 µm after swelling, despite input widths of 0.2–0.6 µm (Figure 4c). A similar trend was observed in additional print fidelity measurements (Figure S8).

Further, several 3D structures with arbitrary shapes (Figure 4d) (including Echinodermania, bone femur, and gyroids) were successfully printed within 2–5 min. It has to be noted that the Echinodermania [38] (Movie S2) has been used as a benchmark to showcase the complexity and precision of the printable features [40, 41]. Compared to previously reported thiol‐ene formulations based on hyaluronic acid (10%–15%) [9], our PVASH formulation enables 2PP microfabrication of complex hydrogel structures at lower polymer concentrations and higher scanning speeds. This feature may open new avenues to future high‐throughput biofabrication applications.

### 2.3 Biocompatibility

**FIGURE 5:**
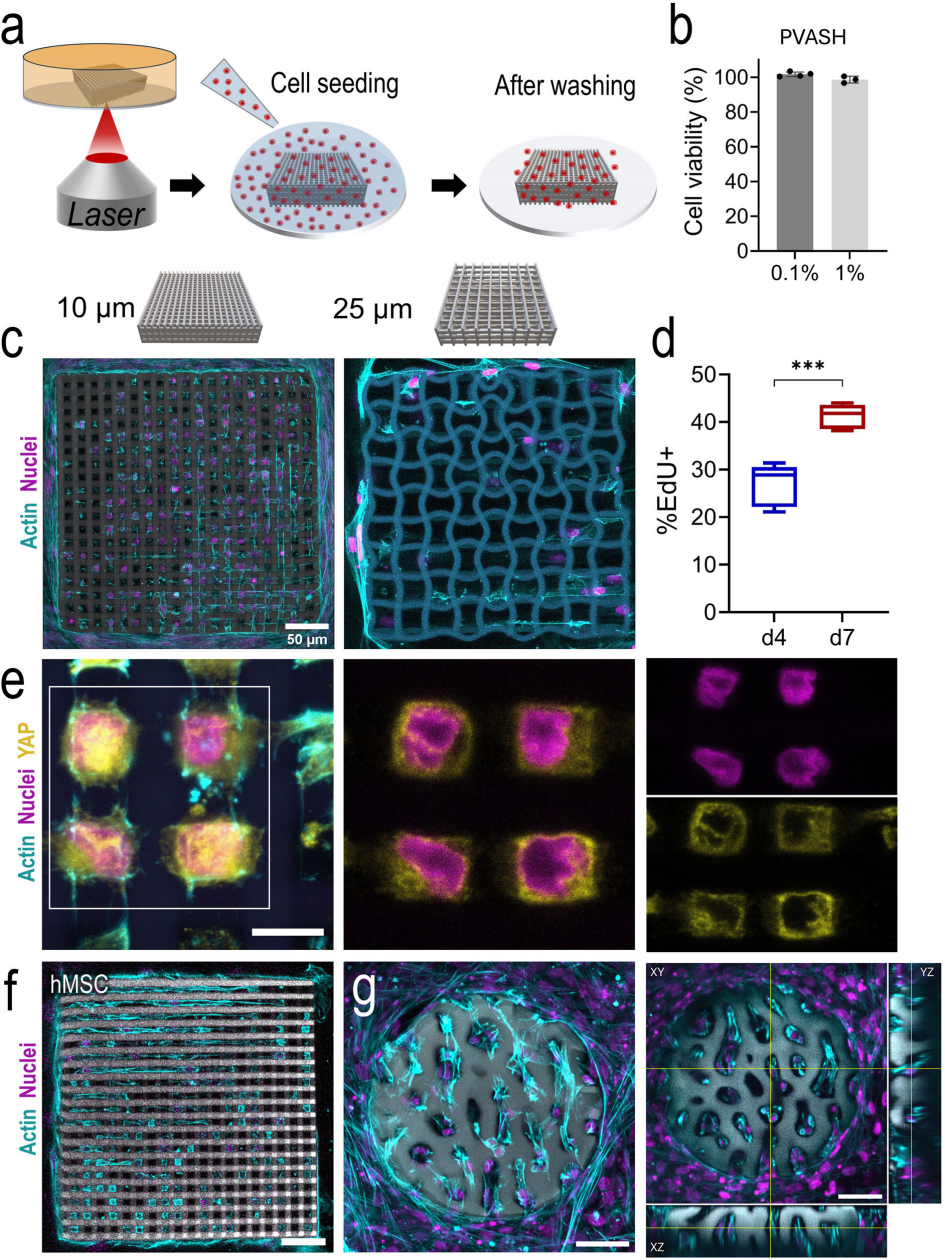
(a) Schematic representation of the cell seeding process on micro‐scaffolds. (b) Cell viability after exposing PVASH solutions with human dermal fibroblasts (HDF) cells for 24 h examined by an MTS cell proliferation assay. (c) Confocal images of actin‐nuclei‐stained HDF on the woodpile micro‐scaffolds (x–y–z: 300 × 300 × 50 µm^3^) of distinct pore sizes (10, 25 µm) for 7 days. Scale bars, 50 µm. (d) Quantification of 5‐ethynyl‐2'‐deoxyuridine (EdU) positive cells at day 4 and day 7. Unpaired two‐tailed *t*‐test, ^***^ *p* = 0.0001, *n* ≥ 5. (e) Confocal images showing actin‐nuclei‐YAP stained HDFs cultured on the 10 µm micro‐scaffolds for 7 days. Scale bar, 10 µm. (f,g) Confocal images showing actin‐nuclei stained hMSC on the woodpile scaffolds at day 7 (f) and femur constructs at day 14 (g). The maximum intensity projection in (g) highlights cell infiltration to the micro‐scaffolds. Scale bars, 50 µm.

Previous studies [32, 33, 42], have confirmed that both nPVA and P2CK are essentially nontoxic. As depicted in Figure 5a, the biocompatibility of PVASH macromer solutions was evaluated using an MTS cell proliferation assay [41, 43]. Human dermal fibroblasts (HDFs) were selected as they are widely used in tissue engineering [44]. Cell viability remained high (> 95%) after incubation with HDF cells for 24 h (Figure 5b). 4% PVASH composition was selected for the fabrication of micro‐scaffolds for cell culture. The hydrogel formulation was chosen for printability and shape fidelity rather than stiffness matching. A series of woodpile micro‐scaffolds with a dimension (x‐y‐z: 300 × 300 × 50 µm^3^) but distinct pore sizes (10, 25, 50 µm) were fabricated (Figure S9). Clickable arginine‐glycine‐aspartic acid (RGD)‐functionalized nPVA conjugates were added into the formulation to promote cell adhesion. Control experiments confirmed that cells did not adhere to micro‐scaffolds lacking RGD, underscoring the necessity of insoluble RGD motifs for cell attachment on microprinted hydrogels (Figure S10). Actin‐nuclei staining was performed to visualize the cytoskeletal organization. Cells exhibited extensive spreading on the micro‐scaffolds. For the 10 µm group, cells were able to grow and spread effectively across the pore space (Figure 5c). Some cells even formed cellular networks through long dendritic protrusions. Notably, some cells exhibited long protrusions and localized cell–cell contacts in response to the micro‐scaffold geometry (Movie S3). In contrast, much fewer cells were found on the 25 µm micro‐scaffolds, presumably because the pore size is too big to retain the cells from sedimentation. An EdU cell proliferation assay was employed to visualize daughter cells after the culture for up to 7 days. The average percentage of EdU+ cells on day 7 appears to be higher than on day 4 (Figure 5d). Additionally, an immunofluorescence staining for the Yes‐associated protein (YAP) showed that most of the YAP signal was confined to the cytoplasm (Figure 5e), indicating that cells perceive a soft environment provided by the micro‐scaffolds.

Human mesenchymal stromal cells (hMSCs) were cultured on the cell‐interactive 10 µm woodpile and trabecular femur constructs for up to 14 days under osteogenic conditions (Figure 5f,g). Besides interacting with the micro‐scaffolds, cells were also observed to contract and cluster with each other, self‐assembling into a dense structure around the scaffolds. This led to heterogeneous cell densities throughout the samples, which may complicate the functional readout. Nevertheless, in some woodpile micro‐scaffolds, mineralization was detected within the pores via OsteoImage staining after two weeks of osteogenic culture (Figure S11). While these findings suggest that hMSCs mineralize their own extracellular matrix within the micro‐scaffolds and successfully differentiate toward the osteogenic lineage, variable cell density remains a limitation of the suspension‐seeding approach. Incorporating a supporting 3D matrix could improve future cultures by promoting homogeneous cell distribution throughout the culture period. These findings confirm the suitability of PVASH hydrogels for 2PP microfabrication of complex cell‐interactive hydrogel structures.

## 3 Conclusion and Future Outlook

In conclusion, we developed a PVA‐based macromolecular thiol‐ene formulation for fast 2PP microfabrication of cell‐interactive hydrogel structures. The macromolecular architecture of the PVASH crosslinker enables efficient thiol‐ene photocrosslinking with nPVA, offering sub‐micron‐scale structural fidelity even at a low precursor concentration of 3%. Our findings highlight the critical influence of molecular architecture on step‐growth 2PP, with PVASH exhibiting superior performance compared to conventional thiol‐ene formulations based on DTT crosslinker [45]. Notably, PVASH maintains consistent printability across a broad concentration range, reducing reliance on high polymer content and facilitating the creation of complex, soft, cell‐interactive hydrogel structures. Despite these promising results, the exact conversion degree of thiol groups after 2PP remains to be tested to quantitatively compare the structural defects in hydrogels of distinct molecular architectures. Moreover, the mechanobiological principles underlying cell‐scaffold interactions as an influence of micro‐geometric cues should be investigated in future work. A deeper understanding of biodegradation and long‐term biocompatibility of the described PVA hydrogel structures may eventually enable geometry‐guided tissue formation both in vitro and in vivo.

## Conflicts of Interest

The authors declare no conflicts of interest.

## Supporting information




**Supporting File 1**: adma72030‐sup‐0001‐SuppMat.pdf

**Supporting File 2**: adma72030‐sup‐0002‐MovieS1.mp4

**Supporting File 3**: adma72030‐sup‐0003‐MovieS2.mp4

**Supporting File 4**: adma72030‐sup‐0004‐MovieS3.mp4

## Data Availability

The data that support the findings of this study are available from the corresponding author upon reasonable request.
