# Supplementary material for: A Water‐Soluble PVA Macrothiol Enables Two‐Photon Microfabrication of Cell‐Interactive Hydrogel Structures at 400 mm s−1
Source: Adv Mater. 2026 Jan 8;38(18):e10834. doi: 10.1002/adma.202510834 (PMC13014024; doi:10.1002/adma.202510834)
Supplement: Supplementary file 1 — Supporting File 1: adma72030‐sup‐0001‐SuppMat.pdf [file ADMA-38-e10834-s003.pdf]

Supporting information

A Water-soluble PVA Macrothiol Enables Two-photon Microfabrication of Cell-interactive Hydrogel Structures at 400 mm s^{-1}

Wanwan Qiu, Margherita Bernero, Muja Emilie Ye, Xianjun Yang, Philipp Fisch, Ralph Müller, and Xiao-Hua Qin*

W. Qiu, M. Bernero, M.E. Ye, X. Yang, P. Fisch, R. Müller, X.-H.Qin
Institute for Biomechanics, ETH Zurich
Gloriastrasse 37/39, 8092 Zurich, Switzerland
Corresponding author: X.-H. Qin (E-mail: qinx@ethz.ch)

W. Qiu, X.-H. Qin
Bringing Materials to Life Initiative, ETH Zurich, Zurich, Switzerland

List of Contents

- Supplementary Figures S1-16
- Supplementary Tables 1-6
- Supplementary Movies S1-3
- References

Experimental section

Materials

All reagents were purchased from Sigma-Aldrich unless otherwise indicated. All reagents were used without further purification except otherwise noted.

Synthesis of Hydrogel Precursors

Norbornene-functionalized PVA (nPVA) Norbornene-functionalized polyvinyl alcohol (nPVA) was prepared as previously reported.[1-2] PVA (8 g, M_W: 47 kDa, cat. no. 10853) was dissolved in anhydrous DMSO (130 mL) under argon protection at 60 °C, and then 15 mL solvent was distilled out under a fine vacuum. Then, p-toluenesulfonic acid (pTsOH, 18.2 mg) was dissolved in anhydrous DMSO (0.5 mL) and transferred into the PVA solution under argon atmosphere. Cis-5-Norbornene-endo-2,3-dicarboxylic anhydride (3.01 g, abcr GmbH) was dissolved in anhydrous DMSO (12 mL) and was added into the PVA/pTsOH solution dropwise. After a 24 h reaction at 55 °C, the solution was cooled down to room temperature and transferred into a dialysis tube (Spectra® M_W cut-off: 6-8 kDa). After dialysis against $NaHCO_3$ (pH 8.0) for 24 h and then against MilliQ water for 24 h, the product was lyophilized to yield 8.7 g of nPVA. The degree of substitution (DS) was 7% as determined by ^{1}H-NMR in D_2O (**Figure S12**).

Thiol-functionalized PVA (PVASH) PVASH was synthesized via a two-step process. Firstly, carboxyl acid groups were introduced into the PVA backbone as described in literature.[3] Briefly, 10 g of PVA (M_W: 27 kDa, cat. no. 81382) was dissolved in 80 mL of anhydrous DMSO under Argon protection at 60 °C. After cooling the solution to room temperature, 3.2 mL of triethylamine and 2.73 g of succinic anhydride were sequentially added. The reaction mixture was then heated to 60 °C and stirred for 24 h. The resulting PVA-COOH was precipitated in ethanol, redissolved in water, and transferred into dialysis tubing (M_W cut-off: 6-8 kDa). Following dialysis against 0.1 M $NaHCO_3$ for 12 h and against MilliQ water for 2 days, the product was lyophilized, yielding ~ 9 g of PVA-COONa. The DS was ~ 10% by ^{1}H-NMR (**Figure S13**).

Secondly, 4 g of PVA-COONa was dissolved in 26 mL of dry DMSO under Argon atmosphere. Then, 715 mg (3.7 mmol, ~0.5 molar equivalents relative to -COOH groups) of N-(3-dimethylaminopropyl)-N′-ethylcarbodiimid-hydrochloride (EDC) and 436 mg (3.8 mmol, ~0.5 molar equivalents) of N-hydroxysuccinimide (NHS) were added sequentially. The mixture was stirred at room temperature for 24 h. Next, 665 mg of cysteamine and 2 g of DTT were added, followed by stirring at room temperature for another 24 h. The reaction mixture was then diluted approx. fivefold with acidic deionized water (pH 3–4) and transferred into dialysis tubing (M_W CO: 6–8 kDa). Dialysis was performed against MilliQ water (pH 3–4) with ~8 water changes over 2 days. The resulting product was lyophilized, yielding ~ 3.6 g of PVASH. The DS was 4.3% as determined by ^{1}H-NMR (**Figure S14**).

RGD-functionalized nPVA (RGDnPVA) RGD-nPVA was prepared by covalent attachment of a cysteine-containing RGD peptide (CGRGDSP, China Peptides) onto the backbone of nPVA through a thiol-ene photoclick reaction.[4] Specifically, 201 mg of nPVA was dissolved in 4 mL of LAP solution (0.05% in PBS). Subsequently, 21 mg RGD peptide was added to the nPVA solution and dissolved under stirring. The mixture was then exposed to a 365 nm LED lamp with an intensity of 70 mW/cm^2 to initiate the thiol-ene reaction. The irradiation was maintained for 1.5 h. Finally, the reaction mixture was transferred into dialysis tubing (M_W cut-off: 6-8 kDa). After dialysis against MilliQ water, the product was lyophilized, yielding 195 mg of RGD-nPVA. ^{1}H-NMR spectrum is shown in **Figure S15**.

Synthesis of P2CK The detailed synthesis of P2CK could be found in a previous report.[5]

Hydrogel Fabrication and Characterization

Study design

Before starting the experiments, we defined polymer concentrations. For the DTT group, we take the content of nPVA as the only contributor to total polymer content, as DTT's molecular weight (154 Da) is negligible. In contrast, the sum of nPVA and thiol crosslinkers is the total polymer concentration for other groups using PEG2SH (2 kDa, Laysan Bio), PEG4SH (20 kDa, , Laysan Bio), and PVASH (35 kDa) group. The weight of the photoinitiator is neglected.

Hydrogel casting

The formulations were prepared by mixing stock solutions of nPVA and thiol crosslinkers in appropriate ratios. Thiol crosslinkers were prepared and used freshly to reach the desired thiol-ene ratio. Similarly, the photoinitiator lithium phenyl-2,4,6trimethylbenzoylphosphinate (LAP) was added to the formulations to reach a final concentration of 0.05%. For photocrosslinking, the precursor solutions were sandwiched between two Sigmacote-treated glass slides separated by multi-well PDMS molds (dimeter: 6 mm) with a gap thickness of 2 mm. Finally, the solutions were exposed to UV-365 nm light (10 mW/cm^2) for 2 min from each side. Detailed formulation information are listed in **Table S3-5**.

Photo-rheology

In situ photo-rheology were carried out on an Anton Paar MCR 302 modular rheometer (Anton Paar GmbH, Graz, Austria) with a 20 mm parallel plate for all groups. A 365 nm UV LED lamp (Thorlabs, Germany) was equipped to induce photopolymerization. UV light was switched on after 1 min of testing. Oscillatory measurements were performed in triplicates (n=3) at 25 °C with a gap thickness of 0.1 mm, 0.5% shear rate and 1 Hz frequency. Mineral oil was added

around the parallel plate to prevent the sample from drying during the testing. The gelation time was calculated by finding the intersection point between the storage (G') and loss (G") modulus after switching the light. The G'-plateau value, the slope of G' (G'-slope) and the time required to reach gelation onset (t_{onset}, G' > 5 Pa) were used as the measures to compare the performance of different photoresins as an influence of different thiol crosslinkers and polymer concentrations.

Mechanical testing

A Zwick material testing machine (Zwick 1456, Ulm, Germany) with a 10 N load cell and parallel-plate compression fixture was used for determining the unconfined compressive modulus of the hydrogel disks. Prior to testing, the cast gels were incubated in PBS for 24 h to reach swelling equilibrium. The diameter and height of each sample were measured with a caliper before the compression test was conducted. The samples were preloaded at 5 mN and subsequently subjected to a compressive strain rate of 0.001 mm s^{-1}. The test ended when the force exceeded 6 N or the strain reached 35%. The compressive modulus (E) was calculated by determining the slope between approximately 10% to 15% deformation from the stress-strain curve. Each group was measured in triplicates (n=3).

Mass swelling ratio

The hydrogel pellets were swollen in PBS for 48 h, and the equilibrium wet mass (M_w) of each sample was recorded on a balance. After lyophilization, the dry mass (M_d) of each sample was recorded. The mass swelling ratio (Q_m) was calculated according to equation 1:

$$Q_m = M_w/M_d \qquad (1)$$

Characterization of hydrogel degradation behavior

Hydrogel discs were fabricated using a 4% PVA formulation (**Table S5**) and subsequently immersed in 2 mL of either 1× PBS (pH 7.4) or 1× PBS (pH 10) at 37 °C. Throughout the incubation course, the buffer solution was refreshed every second day. The compressive modulus and wet weight of the swollen gels were measured at regular intervals, using 3-4 gels per time point.

Thiol conversion measurement

Thiol conversion was quantified using Ellman's reagent. Ellman's reagent was dissolved in 0.1 M phosphate buffer (pH 8.0) at a final concentration of 1 mg/mL. To determine the thiol content

before crosslinking, an aliquot of the resin was diluted with the prepared Ellman's stock solution and incubated at room temperature for 20 min. For post-crosslinking measurements, gels were cast (50 µL per disc), incubated in the Ellman's solution, and homogenized using a pellet pestle motor prior to measurement. Absorbance was recorded at 412 nm using a microplate reader for both pre- and post-crosslinking samples.

The percentage of thiol conversion was calculated according to the following equation:

$$\text{Conversion (\%)} = (A_{\text{before crosslinking}} - A_{\text{after crosslinking}})/(A_{\text{before crosslinking}}) \times 100$$

Two-photon microprinting

The photoresin formulations used for two-photon microprinting are shown in **Table S6**. A water-soluble two-photon photoinitiator (P2CK[6], 2 mM) was used. In addition, 50 ppm pyrogallol was added as a radical scavenger to prevent uncontrolled photopolymerization before, during, and after printing.[7] The impact of polymer concentration (2%, 3%, 4%, and 5%) and type of thiol crosslinker architecture (PVASH, controls: DTT, PEG2SH, and PEG4SH) on printability was investigated. Photoresins were loaded into a PDMS mold (dimeter: 5 mm, thickness: 0.5 mm), a glass coverslip was put atop to prevent from evaporation during printing.

A NanoOne 1000 (UpNano GmbH, Vienna, Austria) equipped with a femtosecond laser at a wavelength of 780 nm (80 MHz repetition, 90 fs pulse length) was used to fabricate 3D microstructures. A 20× water immersion objective (NA = 0.7) was employed for most experiments. The layer height and line distance of the laser were 0.5 and 0.3 µm, respectively. After printing, PBS was added to remove the unpolymerized resin. After 24 h, the fidelity of 3D gel structures was evaluated by confocal imaging on a Lecia SP8-MP microscope equipped with a 25× water-immersion objective (NA = 0.95). A 40× oil-immersion objective (NA = 1.4) was employed to print the line structures in Fine mode. For quantification of line widths, the samples were imaged with a 63× oil-immersion objective (NA = 1.4). The acquired confocal images were analyzed to determine the average line widths of the polymerized structures. For indentation testing, larger cubic samples were printed using a 5× air-immersion objective (NA = 0.25) at a writing speed of 900 mm s^{-1} and a laser power of 290 mW. Prior cell seeding experiments, the samples fabricated with the 20× objective were sterilized by exposure to UVC irradiation for 20 min.

Indentation testing

Bioindentation tests were performed on a UNHT^3 Bio Bioindenter (AntonPaar) equipped with a spherical ruby indenter (diameter: 1 mm). To meet the dimensional requirements of the

instrument, samples were printed using a 5× air-immersion objective (NA 0.25), yielding dimensions of approximately 1.2 × 1.2 × 0.7 mm^3. The samples were printed with the formulation of 4% PVA on glass slides and incubated in PBS for 24 h prior to testing. For indentation, the glass slides were fixed to Petri dishes using super glue, and a small amount of PBS was maintained around the samples to prevent drying. The sample surface was detected by approaching until a load of 10 μN was reached. Samples were then indented to a depth of 60 μm within 5 s, held for 10 s, and subsequently unloaded. The Hertz modulus (E_{Hz}) was calculated using the Indentation software (Indentation v8.0.15, AntonPaar). To directly compare the mechanical properties of printed and cast gels, indentation tests on cast samples were performed under the same conditions as those used for printed gels.

MTS cytotoxicity assay of PVASH

To test the cytotoxic effect of PVASH, a MTS (3-(4,5-dimethylthiazol-2-yl)-5-(3-carboxymethoxyphenyl)-2-(4-sulfophenyl)-2H-tetrazolium) (Abcam, no. 1028026-3) cell proliferation assay was used. Human dermal fibroblasts (HDF) were seeded in a 96-well plate with a concentration of 10 k cells per well and cultivated for 24 h. Cells were treated by replacing the medium containing 0.1% and 1% PVASH solution for 24 h at 37 °C, 5% CO_2. Subsequently, the treatment solution was replaced with MTS (1:10 dilution) testing solution and incubated for 4 h at 37 °C, 5% CO_2. The absorbance of the samples at 490 nm was collected on a plate reader (Tecan). The cell viability (V_R) was calculated according to equation 2:

$$V_R = A/A_0 \times 100\% \tag{2}$$

where A and A_0 are the absorbance of the experimental groups and control group, respectively.

Characterization of RGD-nPVA conjugates

The degree of substitution (DS) of RGD-functionalized nPVA (RGD-nPVA) was determined by comparing the norbornene signal in the ^{1}H NMR spectrum before and after RGD conjugation (**Figure S15**). For instance, the DS of nPVA was 7%, which decreased to 6.2% following RGD grafting, corresponding to a RGD substitution of 0.8%.

For hydrogel formulations in cell culture, 50% of the nPVA fraction was replaced with RGD-nPVA. Under these conditions, the thiol–ene stoichiometric ratio was approximately 100:96. Rheological analysis indicated that incorporation of RGD–nPVA did not significantly alter the mechanical behavior of the hydrogels (**Figure S16**).

Cell seeding on micro-scaffolds

The glass substrates with 3D micro-scaffolds were UV-sterilized. 50 µL of a cell suspension (5 × 10^6 cells/mL) was dispensed into the PDMS mold (diameter = 5 mm, height = 0.5 mm). After incubation for 1 hour, unadhered cells were gently washed away using warm PBS. Subsequently, either normal or osteogenic medium was added to the micro-scaffolds to maintain cell culture. For cell proliferation testing, EdU (10 µM) was added to the culture medium.

Human mesenchymal stem cell (hMSC) culture

Human mesenchymal stem cells (Lonza, PT-2501) were expanded in expansion medium (DMEM, 10% fetal bovine serum (FBS), 1% antibiotic-antimycotic (Anti-Anti), 1% non-essential amino acids and 0.001% bFGF) and passaged before reaching confluence. After cell seeding onto the scaffolds, cell differentiation was initiated by switching to osteogenic medium (DMEM, 10% FBS, 1% Anit-Anti, 50 µg/ml ascorbic acid, 100 nM dexamethasone, 10 mM beta-glycerophosphate). Medium was changed three times per week and cultures were maintained up to 14 days.

Cellular imaging and YAP immunostaining

Cell morphology was evaluated at different time points after fixation with 4% paraformaldehyde (PFA) for 20 min at room temperature. Cell membranes were permeabilized with 0.2% Triton-X100 in PBS for 10 min and unspecific binding sites were blocked with 1% bovine serum albumin (BSA) for 2 h in PBS. Samples were washed with 0.1% BSA in PBS for three times. To visualize nuclei and the actin cytoskeleton, cells were stained with Hoechst 33342 (1:400) and Phalloidin-647 (1:400) in PBS containing 0.1% BSA for 2 hours at room temperature.

For YAP staining, samples were first blocked as described above, then incubated overnight at 4 °C with a primary rabbit anti-YAP antibody (1:50) diluted in PBS containing 0.1% BSA. After three washes with PBS, samples were incubated with a secondary donkey anti-rabbit antibody conjugated to Alexa Fluor 647 (1:200), Phalloidin-AF555 (1:400), and Hoechst 33342 (1:400) in PBS with 0.1% BSA for 2 h at room temperature. Finally, the samples were washed with PBS and imaged using a Leica SP8 confocal microscope equipped with a 25× objective.

For OsteoImage staining, osteogenic hMSC cultures were fixed and stained for actin and nuclei as described above. Additionally, OsteoImage Mineralization Assay reagent (Lonza, PA-1503) was added to the staining solution at a dilution of 1:100. Samples were imaged with

an Andor BC43 spinning disc confocal microscope using a 10× objective. Although the OsteoImage fluorescence overlaps with the P2CK signal within the scaffolds, this weaker background signal could be removed by appropriate thresholding.

The residual photoinitiator or fragmentation products in the printed samples are known to produce autofluorescence that partially overlaps with the emission spectra of common fluorescent dyes such as Alexa-488 and Alexa-555.

EdU cell proliferation assay

To quantify cell proliferation on micro-scaffolds, the samples were stained for EdU according to the protocol by the supplier (Thermo Fisher, no. C10638). Briefly, the fixed samples were washed with 3% BSA in PBS twice and subsequently permeabilized with 0.5% Triton X-100 for 20 min at room temperature. Then, the samples were incubated with EdU staining solution for 30 min. Afterwards, the samples were washed with 3% BSA, and the cell nuclei were stained with Hoechst 33342 (1:2000) in PBS for 30 min at room temperature. Finally, samples were washed with PBS three times and imaged on a Leica SP8 confocal microscope. The percentage of EdU positive cells in total number of cells was determined by manual counting ($n \geq 5$).

Statistical analysis

Results were reported as mean ± standard deviation (SD). Statistical analyses were conducted using GraphPad Prism 10.3.1. All test methods and p values are provided in the figure captions.

Supplementary Figures

Figure S1

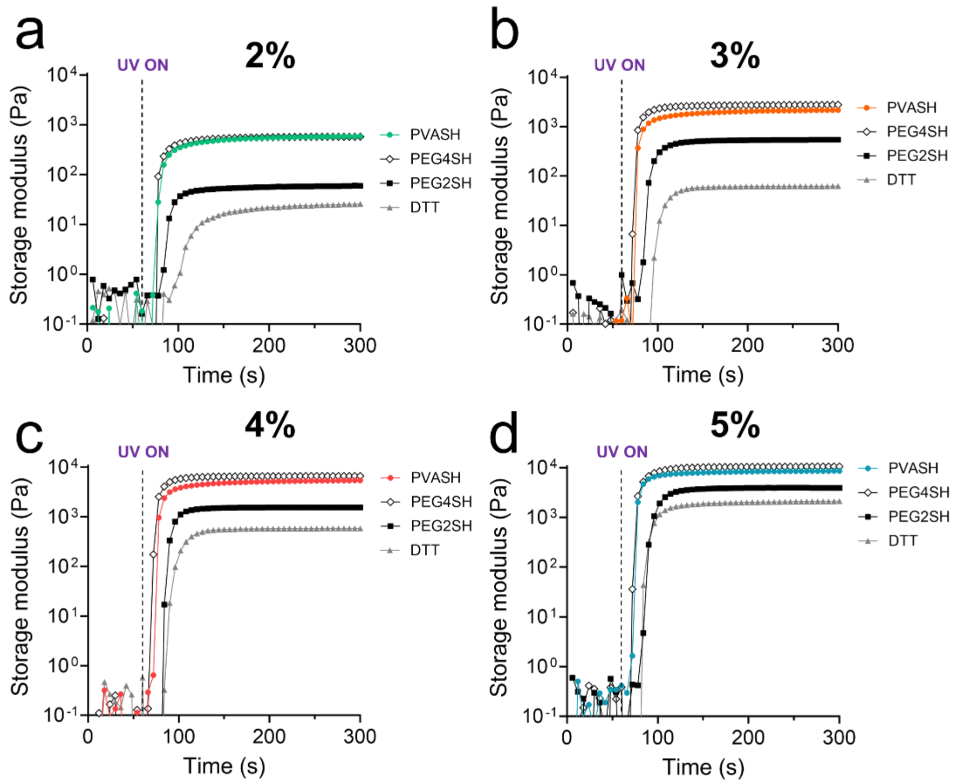

Figure S1. The storage moduli of photoresins of different thiol crosslinkers (PVASH vs. PEG4SH vs. PEG2SH vs. DTT) at different polymer concentrations: 2% (**a**), 3% (**b**), 4% (**c**) and 5% (**d**). The results emphasize the PVASH group has the highest efficiency in thiol-ene photocrosslinking (stoichiometric ratio of 1:1). Light intensity: 10 mW/cm^2.

Figure S2

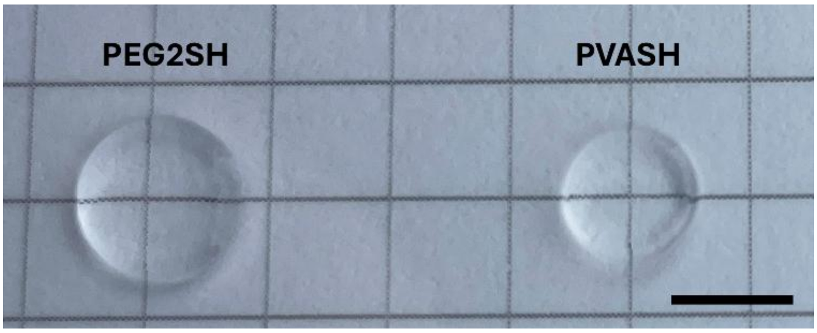

Figure S2. Images showing the physical appearance of PEG2SH and PVASH hydrogels (2%). Scale bar, 5 mm.

Figure S3

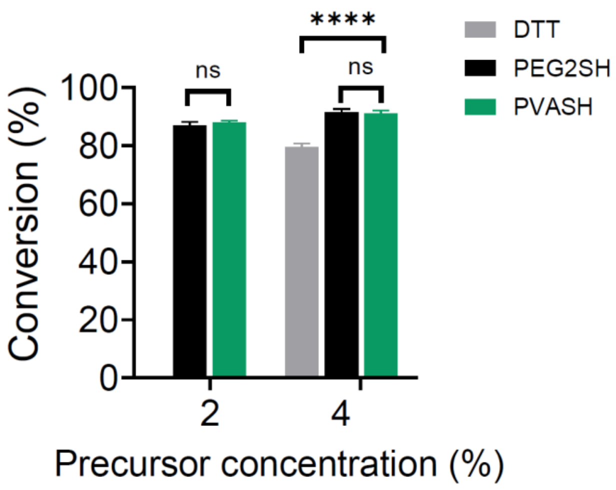

Figure S3. Thiol group conversion of different hydrogel formulations measured by Ellman's assay. Two-way ANOVA. ns, not significant, ****$p < 0.0001$.

Figure S4

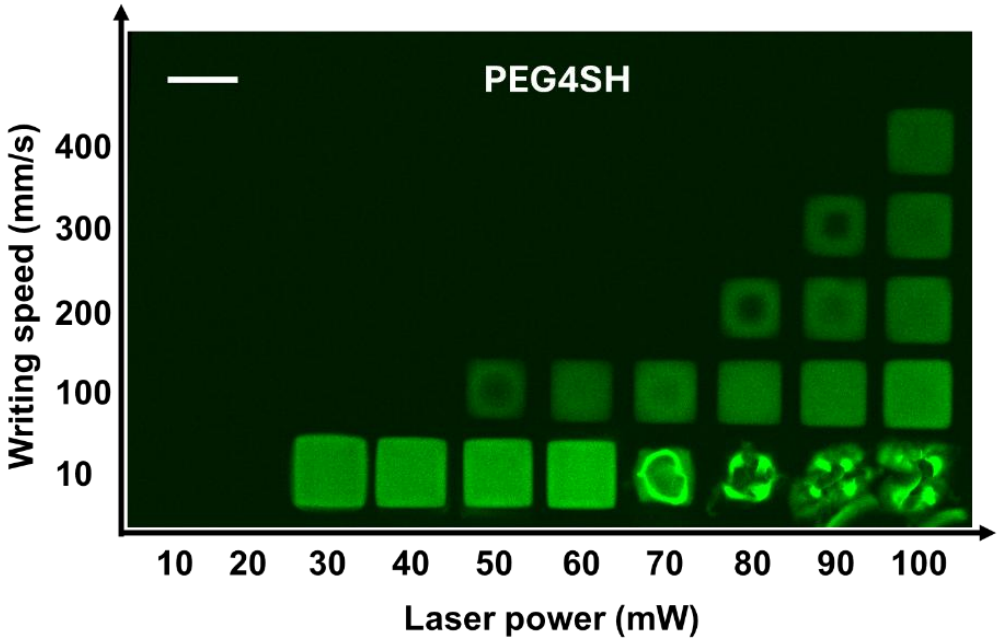

Figure S4. Processing window of 4% formulations with PEG4SH (20 kDa) as the crosslinker at varying laser power and scanning speed. Scale bar, 50 µm.

Figure S5

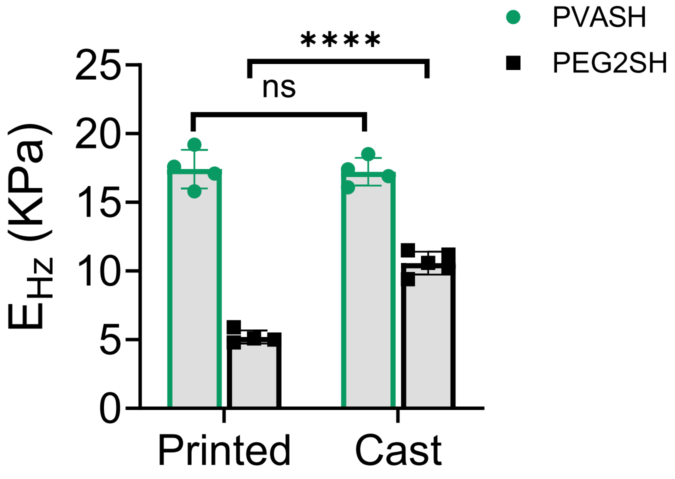

Figure S5. Hertz modulus (E_{Hz}) of the printed and cast gels. Data are presented as mean ± SD ($n \geq 3$). ns, not significant, ****$p < 0.0001$. The samples were fabricated using a 5× air-immersion objective (NA = 0.25) at a writing speed of 900 mm s^{-1} and a laser power of 290 mW.

Figure S6

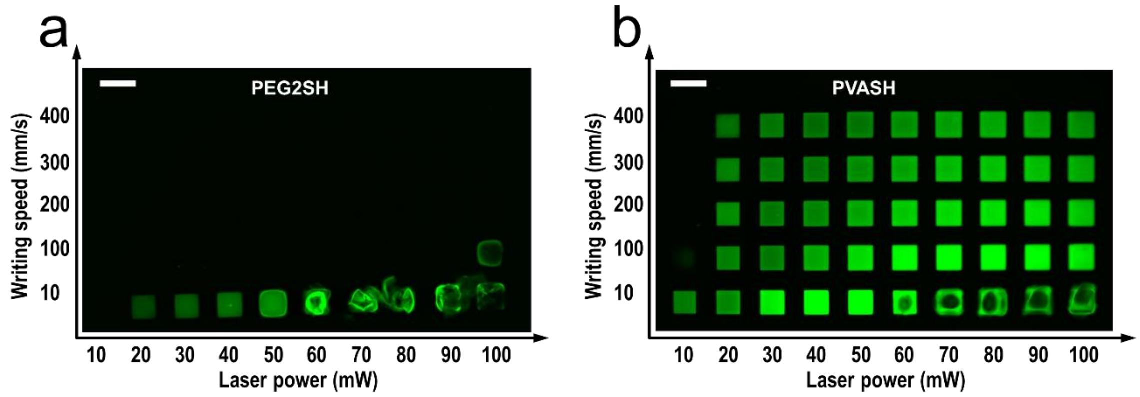

Figure S6. 2PP processing window of the 3% PEG2SH (**a**) and 3% PVASH (**b**) formulations. Scale bars, 50 μm.

Figure S7

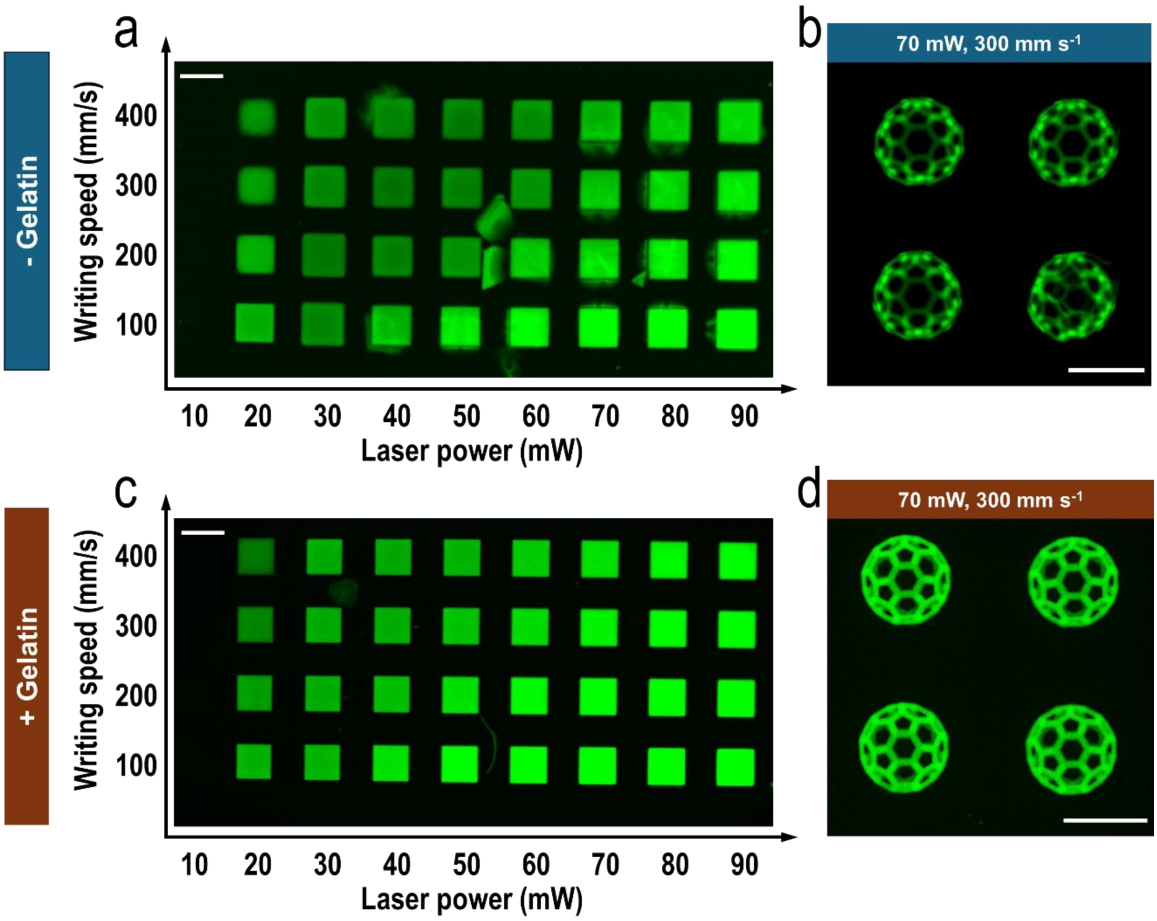

Figure S7. (**a**) 2PP processing window of the 2% PVASH group. **(b)** Maximum intensity projection confocal image of a C_{60} microstructure produced by 2PP with a scanning speed of 300 mm/s and laser power of 70 mW. **(c)** 2PP processing window for the 2% PVASH group by adding 3% sacrificial gelatin. **(d)** Maximum intensity projection image of the gelatin-supplemented PVA microstructures at same printing conditions. Scale bars, 50 µm.

Figure S8

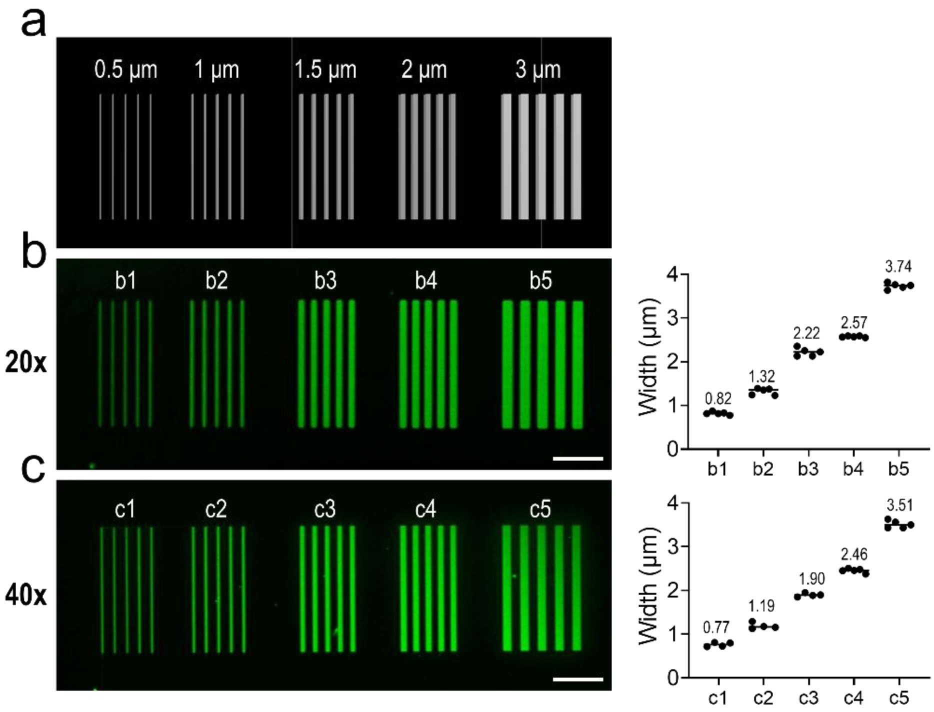

Figure S8. (**a**) Schematic of thin-line models with varying widths for 2PP. (**b-c**) Confocal images of hydrogel lines fabricated using a 4% PVASH formulation, acquired with a 20× objective (**b**) and a 40× objective (**c**), along with corresponding quantitative analysis of line widths. Confocal imaging was performed using a 63× oil-immersion objective. Scale bars, 20 μm.

Figure S9

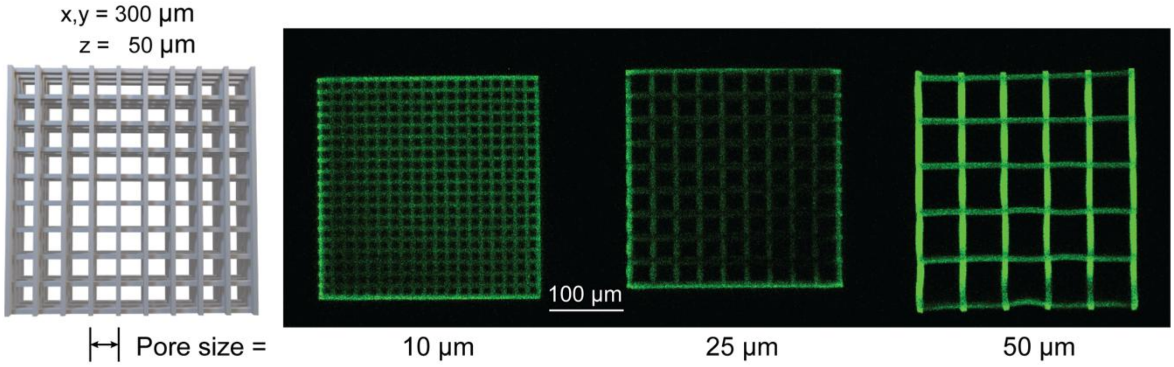

Figure S9. CAD illustration and confocal images of woodpile micro-scaffolds with different pore sizes. Scale bar, 100 µm.

Figure S10

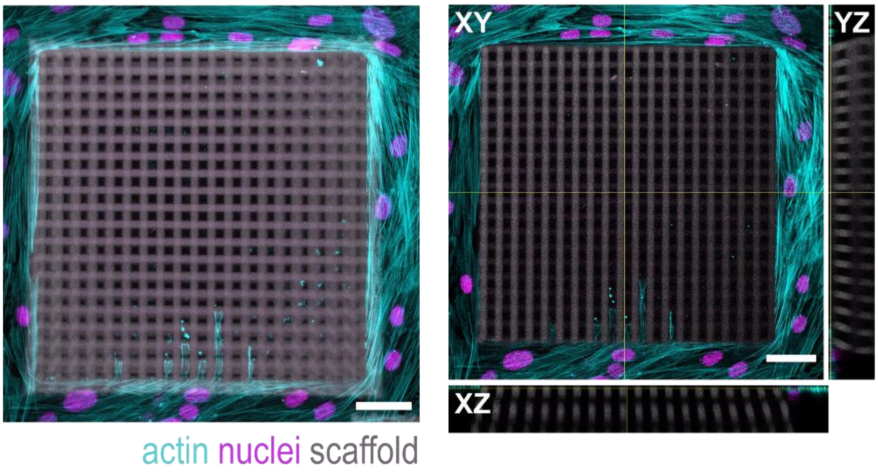

Figure S10. Confocal images of actin-nuclei-stained HDFs on woodpile micro-scaffolds lacking RGD at day 7. Scale bars, 50 μm.

Figure S11

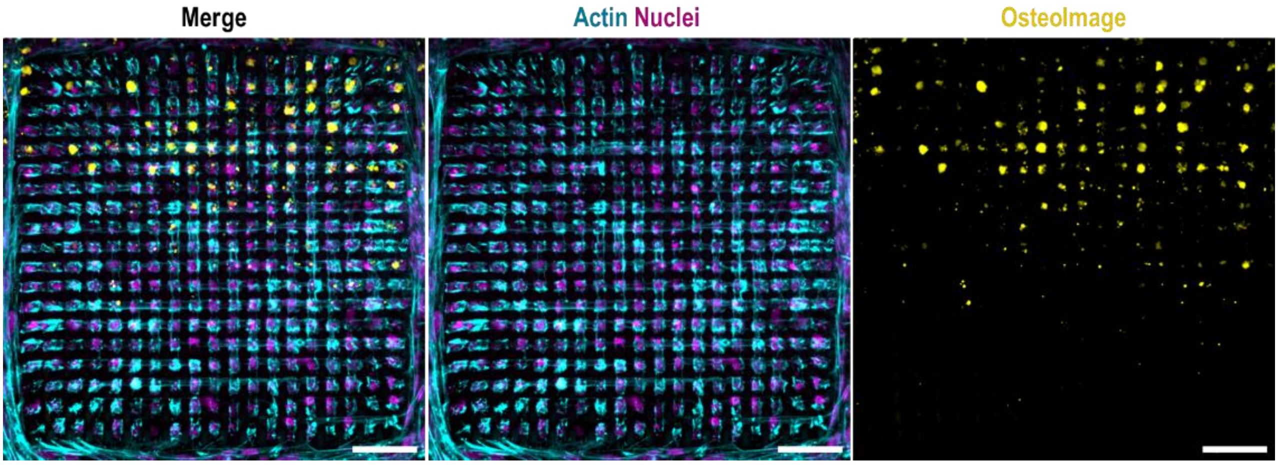

Figure S11. Confocal images (Maximum intensity projection) showing hMSC on the woodpile micro-scaffolds following osteogenic differentiation for 14 days, stained for actin, nuclei and hydroxyapatite (OsteoImage). Scale bars, 50 µm.

Figure S12

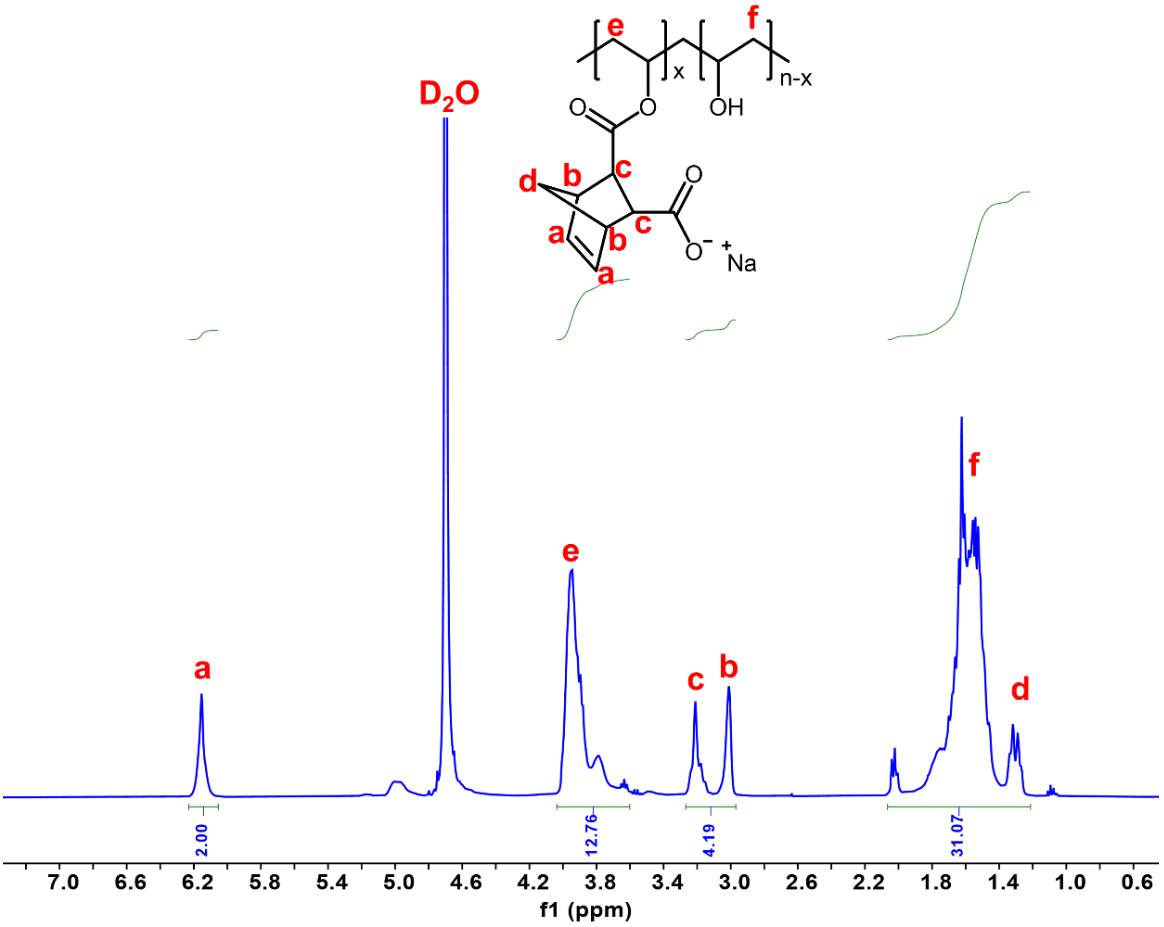

Figure S12. ^{1}H-NMR spectrum of nPVA measured in D_2O. Degree of substitution (DS) is 7%.

Figure S13

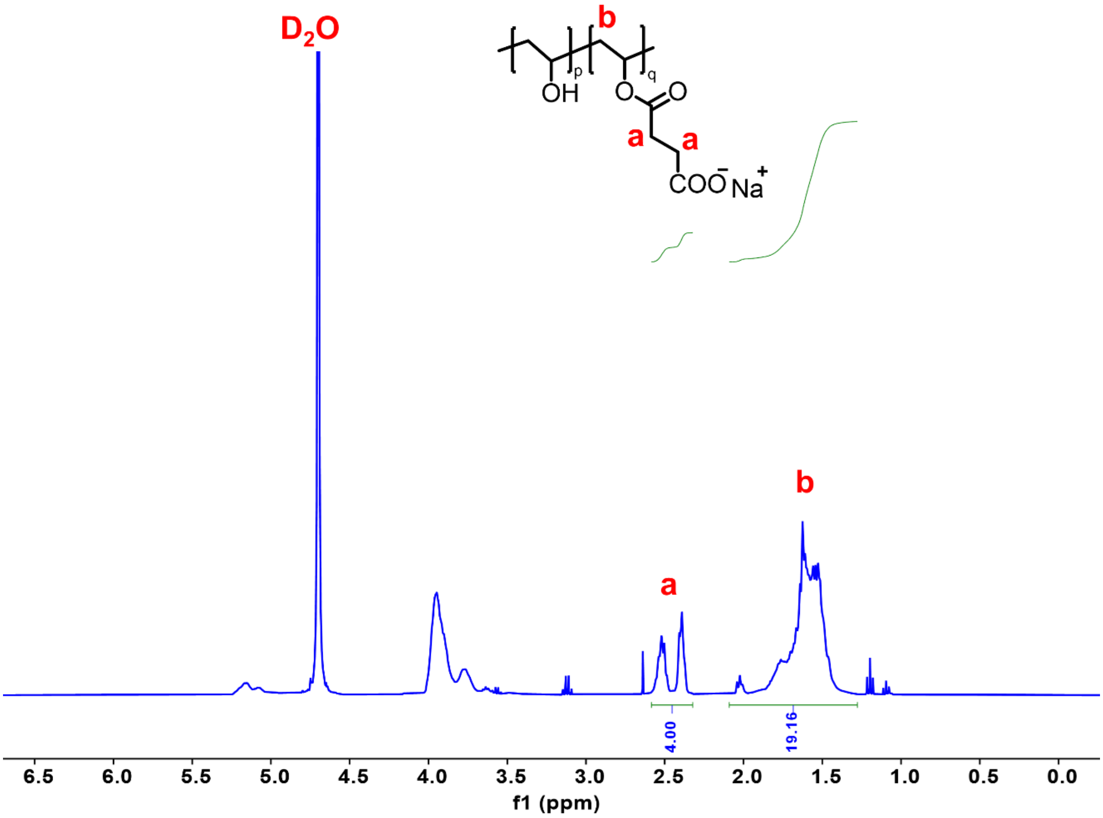

Figure S13. ^{1}H-NMR spectrum of PVA-COONa measured in D_2O. The DS is 9.6%.

Figure S14

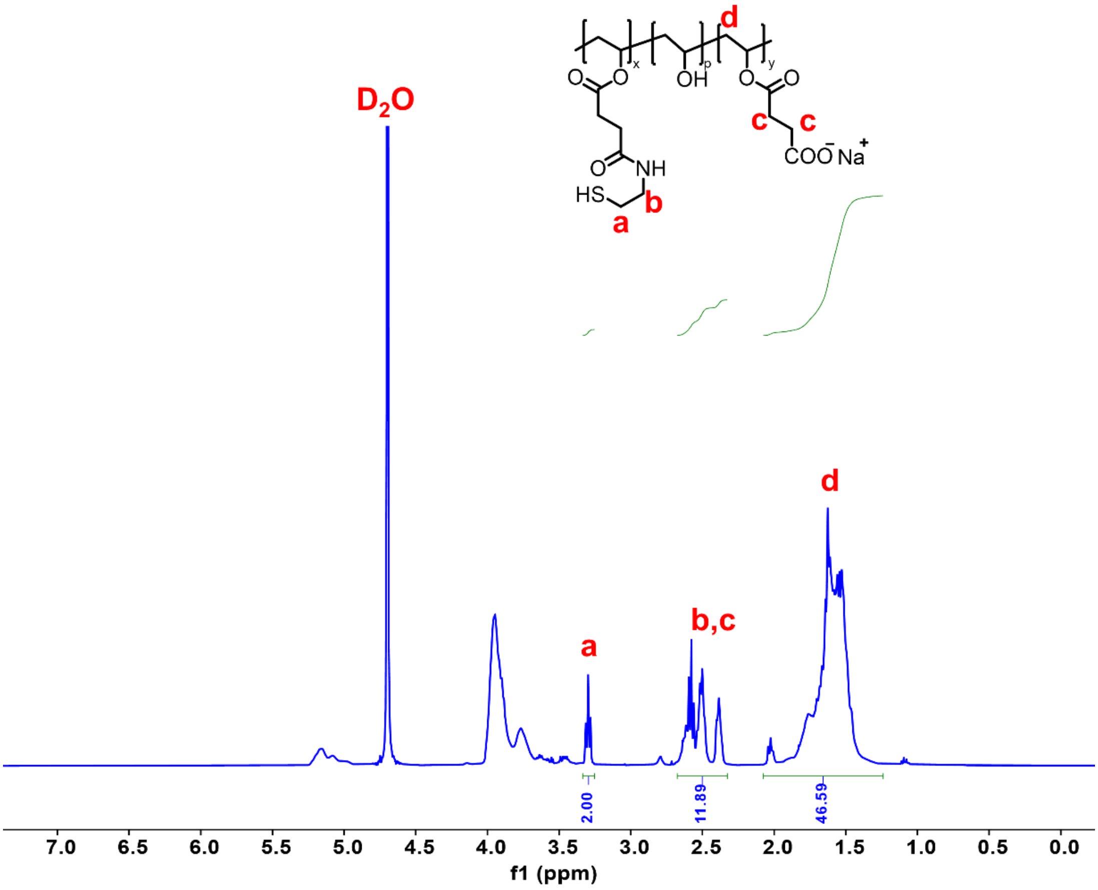

Figure S14. ^{1}H-NMR spectrum of PVASH measured in D_2O. The DS is 4.3%.

Figure S15

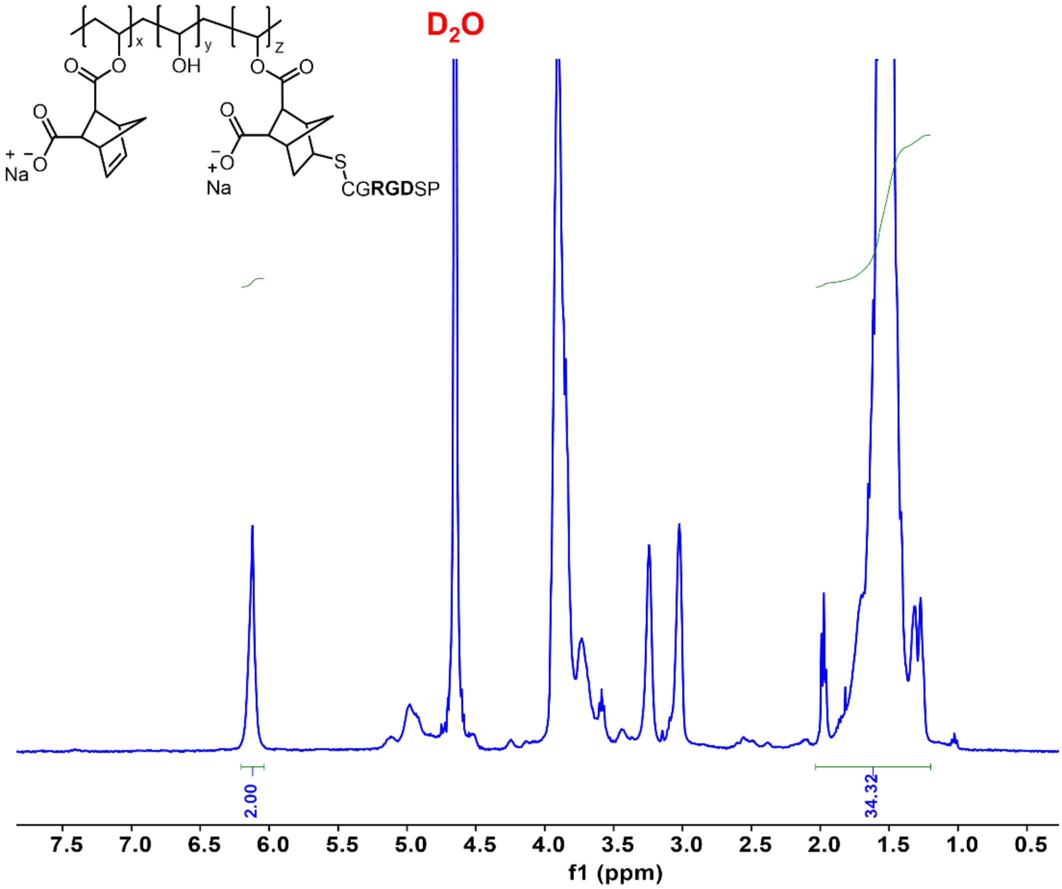

Figure S15. ^{1}H-NMR spectrum of RGD-functionalized nPVA (RGD-nPVA) measured in D_2O. The degree of substitution (DS) of RGD is 0.8%, as determined by comparing the integrals of the norbornene protons before and after RGD conjugation.

Figure S16

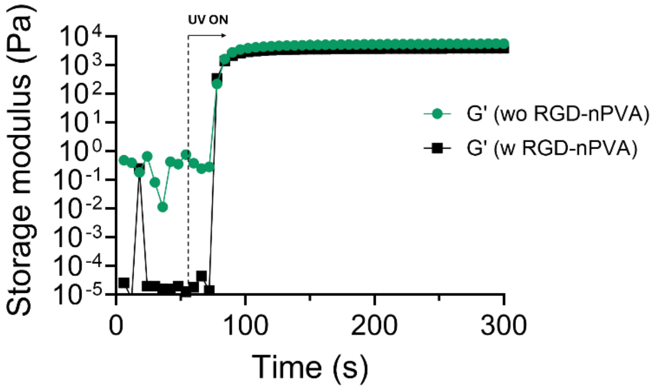

Figure S16. Storage modulus (G′) of the 4% PVASH formulation in the absence (green) and presence of RGD-nPVA (black).

Supplementary Tables

Table S1. Thiol group conversion at a total hydrogel precursor concentration of 2% and 4%, respectively.

	2% PEG2SH	2% PVASH	4% DTT	4% PEG2SH	4% PVASH
-SH conversion (%)	86.0 ± 0.3	85.5 ± 0.2	76 .1± 0.3	87.8 ± 0.4	86.8 ± 0.4

Table S2. Comparison of different photoresins for 2PP nanofabrication and their key parameters.

**Materials**	**Crosslinking mechanism**	**Writing speed mm s^{-1}**	**Objective**	**Macromer content (%)**	**Water content (%)**	**Ref.**
PEGDA	Chain-growth	100	20×	50	50	[6]
GelMA	Chain-growth	7	20×	20	80	[8]
GelNB	Thiol-ene	100	32×	10	90	[9]
GH-VE/BSA thiol	Thiol-ene	50	20×	25	75	[10]
HA-VE/DTT	Thiol-ene	56	20×	20	80	[11]
BSA	Di-tyrosine crosslinking	0.003	100×	40	60	[12]
Silk fibroin/$AgNO_3$	Di-tyrosine crosslinking	2	60×	2.5	97.5	[13]
nPVA/PVASH*	Thiol-ene	50	20×	20	80	[7]

* Note that the study by Baudis et al. shows that their PVASH (one-step synthesis) was inferior to DTT for 2PP, presumably due to the poor water-solubility of the thiol crosslinker.

Table S3. Recipe of nPVA + DTT (thiol-ene stoichiometric ratio of 1:1) at different polymer concentration.

nPVA + DTT									
Material	Stock solution [%w/v]	Volume of stock solution needed for 100 μl [μl]							
		Polymer concentration [%w/v]:	1	1.25	1.5	2	3	4	5
nPVA	10		10.0	12.5	15	20	30	40	50
DTT	5		1.9	2.37	2.85	3.8	5.7	7.6	9.5
LAP	0.5		10.0	10.0	10.0	10.0	10.0	10.0	10.0
PBS			78.1	75.13	72.15	66.2	54.3	42.4	30.5

Table S4. Recipe for nPVA + PEG2SH (thiol-ene stoichiometric ratio of 1:1) at different polymer concentration.

nPVA + PEG2SH									
Material	Stock solution [%w/v]	Volume of stock solution needed for 100 μl [μl]							
		Polymer concentration [%w/v]:	1	1.25	1.5	2	3	4	5
nPVA	10		4.5	5.61	7.0	9.0	13.6	17.6	23.0
PEG2SH	10		5.54	6.91	8.62	11.08	16.75	21.67	28.33
LAP	0.5		10.0	10.0	10.0	10.0	10.0	10.0	10.0
PBS			79.96	77.48	74.38	69.96	59.65	50.73	38.67

Table S5. Recipe for nPVA + PVASH (thiol-ene stoichiometric ratio of 1:1) at different polymer concentration.

nPVA + PVASH									
Material	Stock solution [%w/v]	Volume of stock solution needed for 100 μl [μl]							
		Polymer concentration [%w/v]:	1	1.25	1.5	2	3	4	5
nPVA	10		3.8	4.46	5.7	7.5	11.4	15.2	19.0
PVASH	5		12.56	14.74	18.83	24.78	37.67	50.23	62.78
LAP	0.5		10.0	10.0	10.0	10.0	10.0	10.0	10.0
PBS			73.64	70.8	65.47	57.72	40.93	24.57	8.22

Table S6. Recipe for nPVA + PVASH (thiol-ene stoichiometric ratio of 1:1) at different polymer concentration for 2PP nanofabrication.

nPVA + PVASH					
Material	Stock solution [%w/v]	Volume of stock solution needed for 100 μl [μl]			
		Polymer concentration [%w/v]:	2	3	4
nPVA [a)]	10		7.5	11.4	15
PVASH	5		24.78	37.67	49.56
P2CK	12 mM		16.67	16.67	16.67
Pyrogallol	0.25		2	2	2
PBS			49	32.26	5.03

a) The micro-scaffolds for cell seeding were fabricated through the inclusion of nPVA-RGD at a 1:1 volume ratio with nPVA. The stock solution of nPVA-RGD had a concentration of 10%.

Supplementary Movies

Movie S1. Comparison of 2PP printing performance between PEG2SH and PVASH at 3% precursor concentration.

Movie S2. Animation of the printed microstructure using the Echinodermania model (4%).

Movie S3. Animation of actin-nuclei-stained cell morphologies (day 7) in response to the geometry of RGD-functionalized micro-scaffolds (4%).

References

[1] X. H. Qin, X. Wang, M. Rottmar, B. J. Nelson, K. Maniura-Weber, Near-Infrared Light-Sensitive Polyvinyl Alcohol Hydrogel Photoresist for Spatiotemporal Control of Cell-Instructive 3D Microenvironments, *Advanced Materials* **2018**, *30*, 1705564.

[2] W. Qiu, J. Gehlen, M. Bernero, C. Gehre, G. N. Schädli, R. Müller, X. H. Qin, A Synthetic Dynamic Polyvinyl Alcohol Photoresin for Fast Volumetric Bioprinting of Functional Ultrasoft Hydrogel Constructs, *Advanced Functional Materials* **2023**, *33*, 2214393.

[3] K. S. Lim, M. H. Alves, L. A. Poole-Warren, P. J. Martens, Covalent incorporation of non-chemically modified gelatin into degradable PVA-tyramine hydrogels, *Biomaterials* **2013**, *34*, 7097-7105.

[4] X. H. Qin, K. Labuda, J. Chen, V. Hruschka, A. Khadem, R. Liska, H. Redl, P. Slezak, Development of Synthetic Platelet-Activating Hydrogel Matrices to Induce Local Hemostasis, *Advanced Functional Materials* **2015**, *25*, 6606-6617.

[5] C. Gehre, W. Qiu, P. Klaus Jager, X. Wang, F. C. Marques, B. J. Nelson, R. Muller, X. H. Qin, Guiding Bone Cell Network Formation in 3D via Photosensitized Two-Photon Ablation, *Acta Biomater* **2023**.

[6] Z. Li, J. Torgersen, A. Ajami, S. Mühleder, X. Qin, W. Husinsky, W. Holnthoner, A. Ovsianikov, J. Stampfl, R. Liska, Initiation efficiency and cytotoxicity of novel water-soluble two-photon photoinitiators for direct 3D microfabrication of hydrogels, *RSC Advances* **2013**, *3*, 15939-15946.

[7] S. Baudis, D. Bomze, M. Markovic, P. Gruber, A. Ovsianikov, R. Liska, Modular material system for the microfabrication of biocompatible hydrogels based on thiol-ene-modified poly(vinyl alcohol), *Journal of Polymer Science Part A: Polymer Chemistry* **2016**, *54*, 2060-2070.

[8] A. Ovsianikov, S. Muhleder, J. Torgersen, Z. Li, X. H. Qin, S. Van Vlierberghe, P. Dubruel, W. Holnthoner, H. Redl, R. Liska, J. Stampfl, Laser photofabrication of cell-containing hydrogel constructs, *Langmuir* **2014**, *30*, 3787-3794.

[9] J. Van Hoorick, A. Dobos, M. Markovic, T. Gheysens, L. Van Damme, P. Gruber, L. Tytgat, J. Van Erps, H. Thienpont, P. Dubruel, A. Ovsianikov, S. Van Vlierberghe, Thiol-norbornene gelatin hydrogels: influence of thiolated crosslinker on network properties and high definition 3D printing, *Biofabrication* **2020**, *13*.

[10] X.-H. Qin, J. Torgersen, R. Saf, S. Mühleder, N. Pucher, S. C. Ligon, W. Holnthoner, H. Redl, A. Ovsianikov, J. Stampfl, R. Liska, Three-dimensional microfabrication of protein hydrogels via two-photon-excited thiol-vinyl ester photopolymerization, *Journal of Polymer Science Part A: Polymer Chemistry* **2013**, *51*, 4799-4810.

[11] X.-H. Qin, P. Gruber, M. Markovic, B. Plochberger, E. Klotzsch, J. Stampfl, A. Ovsianikov, R. Liska, Enzymatic synthesis of hyaluronic acid vinyl esters for two-photon microfabrication of biocompatible and biodegradable hydrogel constructs, *Polym. Chem.* **2014**, *5*, 6523-6533.

[12] B. Kaehr, J. B. Shear, Multiphoton fabrication of chemically responsive protein hydrogels for microactuation, *Proc Natl Acad Sci U S A* **2008**, *105*, 8850-8854.

[13] Y. L. Sun, Q. Li, S. M. Sun, J. C. Huang, B. Y. Zheng, Q. D. Chen, Z. Z. Shao, H. B. Sun, Aqueous multiphoton lithography with multifunctional silk-centred bio-resists, *Nat Commun* **2015**, *6*, 8612.
